# HiNT: a computational method for detecting copy number variations and translocations from Hi-C data

**DOI:** 10.1186/s13059-020-01986-5

**Published:** 2020-03-23

**Authors:** Su Wang, Soohyun Lee, Chong Chu, Dhawal Jain, Peter Kerpedjiev, Geoffrey M. Nelson, Jennifer M. Walsh, Burak H. Alver, Peter J. Park

**Affiliations:** Department of Biomedical Informatics, Harvard Medical School, Boston, MA USA

**Keywords:** Chromosomal interactions, Structural variation, Whole-genome sequencing, Repetitive region

## Abstract

The three-dimensional conformation of a genome can be profiled using Hi-C, a technique that combines chromatin conformation capture with high-throughput sequencing. However, structural variations often yield features that can be mistaken for chromosomal interactions. Here, we describe a computational method HiNT (Hi-C for copy Number variation and Translocation detection), which detects copy number variations and interchromosomal translocations within Hi-C data with breakpoints at single base-pair resolution. We demonstrate that HiNT outperforms existing methods on both simulated and real data. We also show that Hi-C can supplement whole-genome sequencing in structure variant detection by locating breakpoints in repetitive regions.

**Electronic supplementary material:**

The online version of this article (10.1186/s13059-020-01986-5) contains supplementary material, which is available to authorized users.

## Background

The Hi-C assay provides genome-wide identification of chromatin interactions, thereby enabling systematic investigation of the three-dimensional genome architecture and its role in gene regulation [1]. Hi-C data have been used, for example, to characterize topologically associated domains (TADs), which are megabase-sized local chromatin interaction domains within which genomic loci interact with higher frequency [2–4]. Characterization of genome organization using Hi-C data has enhanced our understanding of a number of biological processes, such as X-inactivation [2, 5], cell cycle dynamics [6], and tumor progression [7].

However, it has been shown that structural variations (SVs) can confound the interpretation of Hi-C data [6, 8–11]. For example, when there is copy number increase, the observed number of sequencing reads that correspond to chromosomal interactions in that region will be larger than expected, not because there is greater frequency of interaction but because there are multiple copies of that region. Similarly, when there is an interchromosomal translocation, the reads that correspond to interactions between the translocated segment and its proximal regions will be inflated, but this should not be mistaken for changes in interaction frequency.

One approach to mitigate the impact of SVs on the Hi-C interaction map is to first identify SVs using whole-genome sequencing (WGS) data and then use that information to adjust the Hi-C map. Although a great deal of progress has been made in WGS-based SV detection [12, 13], the use of WGS data requires additional sequencing and analysis expertise. Furthermore, SV breakpoints within repetitive regions, which are often genomic SV hotspots, cannot be easily detected from WGS due to low mappability [14]. Indeed, Hi-C and WGS data are complementary in SV detection: as Hi-C read pairs span genomic distances from base pairs to megabases, they enable detection of breakpoints in repetitive regions when one read of a read pair maps to a repetitive region and the other maps to a surrounding mappable region (Additional file 1: Fig. S1).

Here, we present HiNT (Hi-C for copy Number variation and Translocation detection), an algorithm for detection of copy number variations (CNVs) and interchromosomal translocations in Hi-C data. Based on simulated data and comparisons to variants identified in WGS, HiNT outperforms existing computational methods both in sensitivity and false discovery rate (FDR). HiNT also provides translocation breakpoints at single base-pair resolution, a feature not available in existing methods that utilize only Hi-C data. Furthermore, HiNT supports parallelization, utilizes efficient storage formats for interaction matrices, and accepts multiple input formats including raw FASTQ, BAM, and contact matrix. HiNT is available at https://github.com/parklab/HiNT.

## Results

### Overview of HiNT

HiNT has three main components. HiNT-PRE performs preprocessing of Hi-C data and computes the contact matrix, which stores contact frequencies between any two genomic loci. HiNT-CNV and HiNT-TL start with a Hi-C contact matrix and predict copy number segments and interchromosomal translocations, respectively (Additional file 1: Fig. S2).

**Fig. 1:**
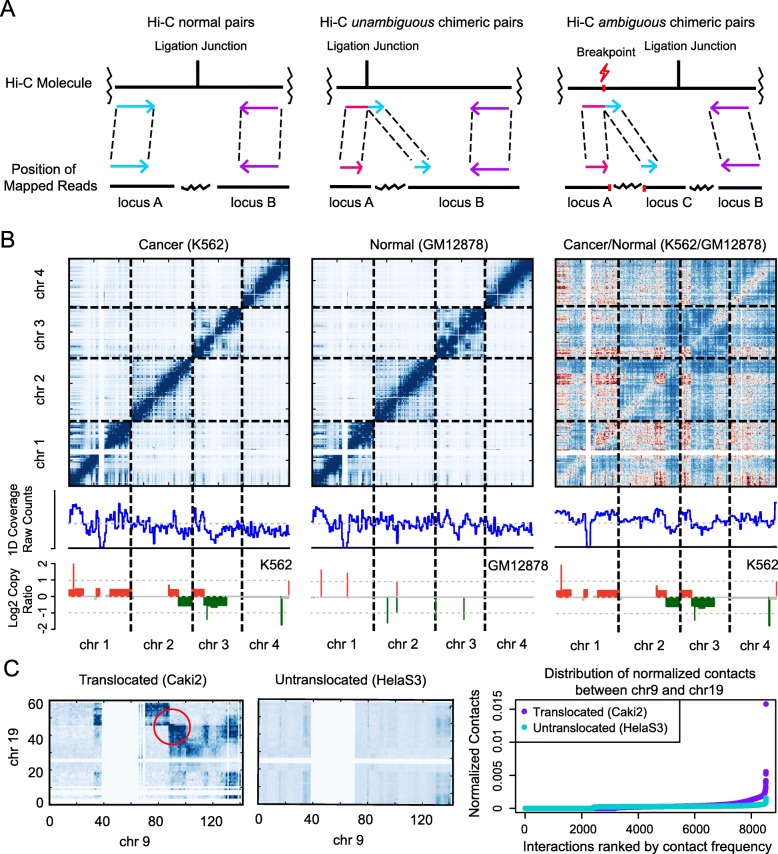
Illustration of HiNT. **a** Hi-C read pairs are classified into normal pairs (left panel), *unambiguous* chimeric pairs (middle panel), and *ambiguous* chimeric pairs (right panel). Hi-C *unambiguous* chimeric pairs are the product of Hi-C ligations in which one read crosses the ligation junction and thus maps to both locus A and locus B, while the other normal read maps only to locus B. Hi-C *ambiguous* chimeric pairs are often caused by structural variations, with one read mapping to both locus A and locus C, while the other read maps to locus B. **b** Copy number information is reflected in the Hi-C 1D coverage profile after Hi-C biases are removed by normalizing the K562 Hi-C contact matrix with the GM12878 Hi-C contact matrix. The copy number profile (log2 ratios) estimated from WGS data is shown in the bottom row for comparison. **c** Comparison of the Hi-C contact matrix between chr9 and chr19 in samples with and without translocations. The distribution of normalized contact frequencies is higher in the sample with translocation (purple dots) than in the sample without (cyan dots). Contact frequencies were calculated in 1-Mb bins in chr9 and chr19

HiNT-PRE aligns read pairs to the genome using BWA-MEM [15] and creates a Hi-C contact matrix. The matrix is constructed from normal read pairs (non-chimeric reads that map uniquely to the genome) as well as *unambiguous* chimeras [16] (Fig. 1a). The latter is a product of Hi-C ligation and is defined as a read pair in which one chimeric read is split into locus A and locus B and the other read is uniquely mapped to locus B (Fig. 1a). All other read pairs containing split reads are defined as *ambiguous* chimeras [16], which will be used for translocation breakpoint detection (Fig. 1a).

HiNT-CNV (Additional file 1: Fig. S2) first creates a one-dimensional (1D) coverage profile across the genome by calculating row or column sums of the contact matrix at a fixed resolution, e.g., 50 kb. These sums should be correlated with the copy number across the bins since they correspond to the strength of interaction of that region with all other regions. It is critical to use the *unnormalized* contact matrix here because the matrix-balancing normalization (setting the sum of each row or column to be 1), which is the most widely used Hi-C normalization approach, removes not only biases but also copy number information. The next step is to perform further adjustment to remove other biases that are inherent in the Hi-C experiments, such as GC content, mappability, and restriction site frequency. In Fig. 1b, we see that, without additional adjustment, the 1D profiles for K562 (human chronic myelogenous leukemia cell line; known to have high genomic instability) and GM12878 (human lymphoblastoid cell line) show similarity to each other but not with the copy number profiles estimated from WGS. However, when we remove Hi-C internal biases in K562 by using GM12878 as a control (Fig. 1b, right), the 1D coverage profile becomes highly correlated with the (ploidy-adjusted) copy ratios estimated from WGS data. This result shows that proper normalization is essential in extracting copy number information from Hi-C data. Given that an appropriate control is often unavailable, HiNT-CNV uses a generalized additive model to remove the biggest sources of bias: GC content, mappability, and restriction fragment length (see Methods) [17, 18]. The boundaries of CNV segments are determined using the BIC-seq segmentation algorithm, which utilizes the Bayesian information criterion to identify regions with enriched or depleted read counts [19]. We used the latest version BIC-seq2 [20] that does not require a matched control. It is important to tune the parameter *λ* in BIC-seq2 to achieve the desired level of smoothness in the CNV profile. Other CNV segmentation algorithms may also be substituted in place of BIC-seq2.

HiNT-TL (Additional file 1: Fig. S2) detects translocations by analyzing normalized interchromosomal interaction matrices. In general, contact probabilities between two regions on the same chromosome decrease monotonically with distance, and interchromosomal interactions are considerably less frequent compared to intra-chromosomal ones. When an interchromosomal translocation occurs, we expect the contact probabilities in two opposite quadrants around the breakpoint to be elevated to the levels observed for adjacent chromosomal regions (Fig. 1c). Thus, HiNT-TL identifies candidate translocated chromosomal pairs based on the presence of high contact probabilities and their unequal distribution. To identify exact breakpoints, HiNT-TL first identifies the breakpoint regions with a coarse 100-kb resolution from the 1D profiles (see “Methods”). HiNT-TL then uses Hi-C *ambiguous* chimeric reads located within these regions to refine breakpoints to single base-pair resolution.

### CNVs predicted by HiNT from Hi-C are consistent with those identified from WGS

To predict CNVs, we first calculate the coverage profile throughout the genome at 50 kb resolution. We then correct for Hi-C biases such as GC content, mappability, and the number of restriction sites (given a fixed bin size, the number of expected fragments depends on the number of cut sites by the restriction enzyme used). To model the non-linear correlation between 1D coverage and biases observed (Additional file 1: Fig. S3), we use a generalized additive model (GAM) with the Poisson link function. GAM is an ideal framework here, as it allows non-parametric fitting with relaxed assumptions on the relationship between predictor and response variables. The copy number information is extracted from regression residuals by the following equation:

$$ \log \left(\mathrm{Coverage}\right)={s}_1\left(\mathrm{GCcontent}\right)+{s}_2\left(\mathrm{Mappability}\right)+{s}_3\left(\mathrm{NumberOfRestr}.\mathrm{Sites}\right)+\varepsilon $$

where *s*_*i* (*i* = 1, 2, 3)_(•) is an unspecified function estimated for each predictor variable and *ε* is the residual. The model fits better for GM12878 (*R*^2^ = 0.798) than for K562 (*R*^2^ = 0.631), since K562 is known to have more SVs.

**Fig. 2:**
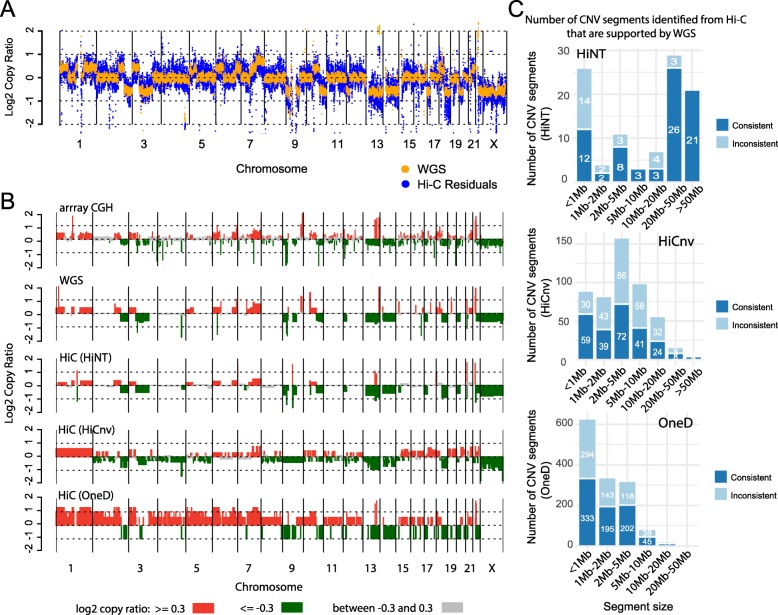
Copy number inference in K562 cells. **a** Comparison of log2 copy ratios calculated using regression residuals from Hi-C (blue) and using read coverage from WGS (orange). **b** Comparison of CNV profiles from Hi-C, array CGH, and WGS after segmentation. Red, green, and gray bars represent copy gain (log2 copy ratio > 0.3), copy loss (log2 copy ratio < − 0.3), and copy neutral regions (log2 copy ratio between − 0.3 and 0.3), respectively. **c** The number of CNV segments (categorized by size) detected from Hi-C that are also supported by WGS. Specifically, 85.91%, 44.74%, and 60.98% of the large CNV segments identified by HiNT, HiCnv, and OneD are supported by those from WGS, respectively. The overlap criteria for consistency are shown in Additional file 1: Fig. S5C

To evaluate CNVs identified from Hi-C, we compare the log2 copy ratios along the genome from the model above with those estimated from WGS. For K562, we see that copy number alterations are prevalent and that the log ratios from Hi-C and WGS are mostly concordant (Fig. 2a, Additional file 1: Fig. S4A; Spearman correlation = 0.82). For GM12878, the correlation is lower (Spearman correlation = 0.21) because there are very few CNVs in this cell line, and the existing small ones are detected only from WGS (Additional file 1: Fig. S4B, Additional file 1: Fig. S5A). The copy ratios fluctuate more in the Hi-C profile relative to WGS data (Fig. 2a, Additional file 1: Fig. S5A) due to the different read depth and possibly due to Hi-C biases that may not have been captured by our model. When the copy number log ratios are segmented using BIC-seq [19], the concordance between the platforms is striking (rows 2 and 3 in Fig. 2b), with ~ 85% and 92% of the large (> 2 Mb) segments from Hi-C overlapping those from WGS in K562 and GM12878, respectively (Fig. 2c, Additional file 1: Fig. S5D; our definition of overlap is described in Additional file 1: Fig. S5C). The copy number profile from array comparative genomic hybridization (CGH) data obtained from Zhou et al. [21] is also mostly concordant (row 1 in Fig. 2b).

To ensure that our results are generalizable, we applied HiNT-CNV to five more cell lines: Caki2 (human renal cancer cell line), LNCaP (human prostate adenocarcinoma cell line), MCF7 (human breast cancer cell line), PANC-1 (human pancreatic cancer cell line), and CHM13hTERT (an effectively haploid cell line, abbreviated as CHM13). Our results show that copy number profiles estimated by HiNT agree well with those inferred from WGS, with a Spearman correlation of ~ 0.8 (Additional file 1: Fig. S6-10A) in most cells. The low correlation (0.4) in LNCaP cells may due to the poor quality of its Hi-C data [22]. More than 80% of large CNV segments identified by HiNT are supported by those identified from WGS in most cell lines (exact overlaps are described in the figure legends of Additional file 1: Fig. S6-10B,C). Collectively, our analysis suggests that HiNT is a reliable tool for identifying large-scale CNVs in both cancer and normal Hi-C data.

### HiNT outperforms HiCnv and OneD for identifying CNVs from Hi-C data

We compared the performance of HiNT to that of two other algorithms. HiCnv [23] infers copy number from normalized Hi-C coverage by employing kernel density smoothing followed by a hidden Markov Model; however, it also requires a baseline chromosome copy number from WGS or karyotyping to determine the true copy number of each chromosome. OneD [24] estimates copy number via a hidden Markov model on the corrected contact frequencies obtained from a generalized additive model. When we compare the copy number profiles generated by HiCnv and OneD to those derived from WGS, we find that they are largely discordant. The Spearman correlations of log2 copy ratios inferred from HiCnv and WGS are 0.67 in K562, 0.1 in GM12878, and 0.03 in CHM13 (Additional file 1: Fig. S4C-F, Additional file 1: Fig. S10A). Moreover, only 44.74%, 27.64%, and 70% of the large CNV segments detected by HiCnv overlap those identified from WGS in K562, GM12878, and CHM13, respectively (Fig. 2b, c, Additional file 1: Fig. S5B,D, Supp. Fig, 10B-C). The concordance between HiCnv and WGS is better in Caki2, LNCaP, MCF7, and PANC-1, but it is still less than that observed for HiNT (Additional file 1: Fig. S6–9; the exact correlations and overlaps have been labeled in the figures or figure legends). For OneD, the copy number log ratios are largely discordant with WGS in all cell lines except CHM13, with the correlation between 0.3–0.5 and only ~ 50% of the large CNV segments agreeing with those inferred from WGS (Fig. 2b, c, Additional file 1: Fig. S4E-F, Additional file 1: Fig. S5–10, the exact correlations and overlaps are in the figures or legends).

In addition, input to HiCnv must be either HiC-Pro [25] output or a SAM file, which is then converted to HiC-Pro format, incurring high computational cost for terabyte-scale datasets. For example, 3 billion read pairs result in a ~ 600 GB BAM file, and the required SAM format is at least fourfold larger than BAM format in size. In contrast, HiNT-PRE accepts FASTQ and BAM files and generates the Hi-C contact matrix in hic [16, 26] or cool [27] format, which serves as the input to HiNT-CNV. Both hic and cool are efficient and widely used formats for genomic interaction matrices. Taken together, HiNT-CNV outperforms these existing tools in detecting CNVs in both cancer and normal cell lines in both accuracy and usability.

### HiNT accurately identifies translocated chromosomal pairs

**Fig. 3:**
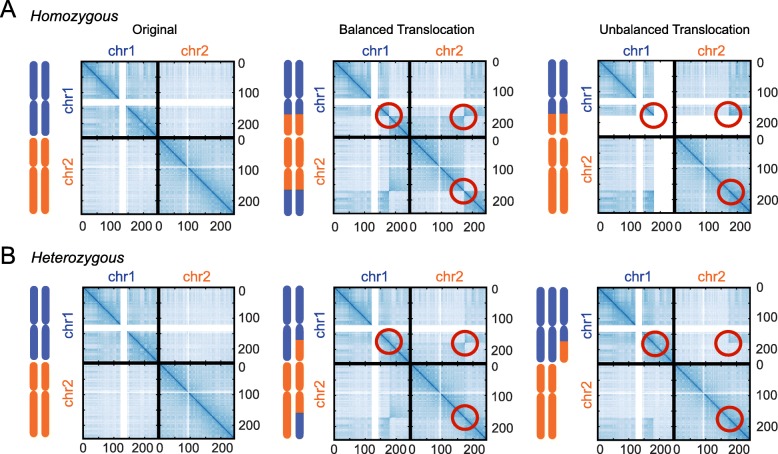
Simulated translocations in Hi-C data. **a** Homozygous cases. **b** Heterozygous cases. An example of a translocation involving two chromosomes is illustrated. The three columns correspond to original matrix, balanced translocation, and unbalanced translocation, respectively. Circles highlight the features introduced by the translocations

Translocations modify the 3D organization of the genome, and they will be incorrectly identified as long-range interactions in Hi-C data if they are not accounted for properly. To first study their impact on Hi-C interaction maps, we developed a simulation scheme to recapitulate the effect of translocations, encompassing homozygous/heterozygous and balanced/unbalanced translocations. A balanced translocation is an even exchange of segments between chromosomes without genetic information gain or loss; an unbalanced translocation involves a loss or gain of chromosome segments. As observed in previous studies [23, 28, 29], a balanced translocation forms a “butterfly” appearance in the chromosomal interaction map (Fig. 3a and Fig. 3b middle, marked by red circles). In contrast, an unbalanced translocation only has a single block (Fig. 3a and Fig. 3b, right column, marked by red circles) [28]. Detection of intra-chromosomal translocations is complicated by the presence of chromatin structures such as TADs and loops. Therefore, we focus on identification of interchromosomal translocations.

Our method is based on detection of two characteristics. First, the contact frequencies should be distributed unevenly around the translocation breakpoint. For this, we utilize the Gini index, a statistical measure of distribution initially used to quantify income inequality in economics [30]. To compute this index, we estimate the cumulative distribution of contact frequencies in each square of the interaction map (we use 1 Mb × 1 Mb) and determine how much it deviates from a linear increase (see “Methods”). A high index corresponds to a more uneven distribution of interaction strength. Second, the maximum interaction level surrounding the breakpoint should be high for a translocation. Regions without a translocation but with a high noise level may satisfy the first criterion of uneven contact frequencies, but their maximum interaction level would not be large. Combining the two features (interaction level and evenness), we define the rank product score as *RP*_*i*_ = (*R*_*gini*,*i*_/*n*) ∗ (*R*_*mif*,*i*_/*n*), where *R*_*gini*,*i*_ and *R*_*mif*,*i*_ are the ranks of matrix *i* based on Gini index and maximum interaction frequency, respectively, and *n* is the total number of interchromosomal interaction matrices.

**Fig. 4:**
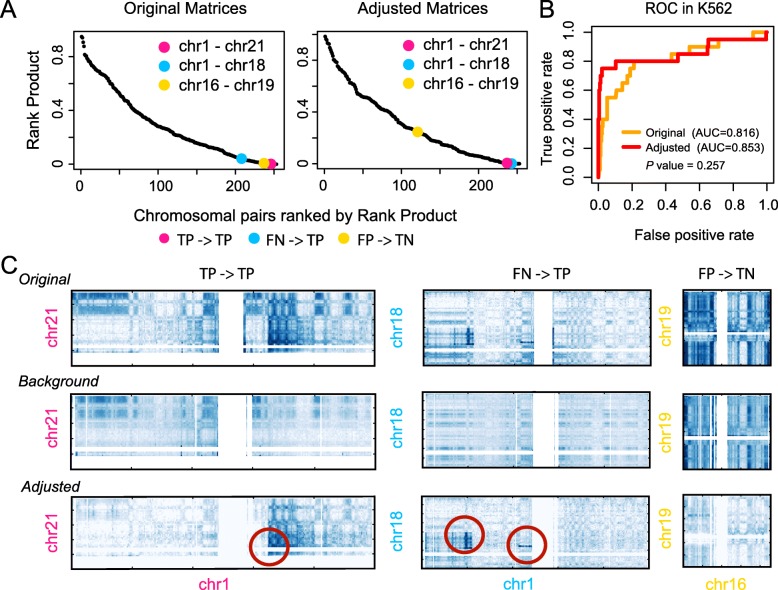
Accurate identification of translocated chromosomal pairs by HiNT. **a** The distribution of the rank product scores for all chromosomal pairs in K562 before (left) and after (right) adjustment by background subtraction. Chromosomal pairs in pink and blue correspond to two FISH-validated translocation pairs (chr1, chr21) and (chr1, chr18); the one in yellow corresponds to a chromosome pair (chr16, chr19) without translocation. After matrix adjustment, the blue pair now has a lower score and the yellow pair has a higher score, as desired. TP: true positive, TN: true negative, FN: false negative, FP: false positive, 0.05 is used as the cutoff. **b** Receiver-operator characteristic (ROC) curves show HiNT performs better after the background subtraction. Areas under the ROC curves (AUCs) are shown in parentheses. **c** The original, background (average of multiple other Hi-C maps), and the adjusted maps are shown for the three cases highlighted in panel **a**. Validated translocations are marked by circles

The rank product score performs well in simulated data, separating the translocated and non-translocated cases in nearly all cases (Additional file 1: Fig. S11). For real data, we found that direct application of the rank product was insufficient, due to the various factors that are not captured by the normalization step, e.g., the A/B compartment effect and the increased interactions between small chromosomes or between sub-telomeric regions. To eliminate such biases, we created a background interaction matrix by averaging the matrices from five normal cell lines (Additional file 2: Table S1, see “Methods”) and used it to normalize the original matrix. In Fig. 4a, we show three examples of chromosomal pairs in K562 data whose scores change as a result of the additional normalization. In the first case (chr1- chr21), the score does not change significantly; in the second case (chr1- chr18), the score increases so that a translocation is now called; and in the third case (chr16 - chr19), the score decreases so that a mistaken call is avoided. Using the chromosomal pairs reported in the literature or validated by FISH experiments [4, 29] as true positives, we see that the adjusted matrix results in an increased prediction accuracy, as measured by the area under the curve (AUC) (Fig. 4b; see “Methods”). As visualized in Fig. 4c, the previously observed biases are effectively reduced by the normalization, allowing for better delineation of translocations (Additional file 1: Fig. S11, Additional file 1: Fig. S12A-D).

Although the rank product approach detects the majority of translocated chromosomal pairs, four validated translocations are not identified. To investigate this issue, we compare the Hi-C interaction matrices of the detected (Additional file 1: Fig. S13) and missed chromosomal pairs (Additional file 1: Fig. S14). Compared to the detected chromosomal pairs, no translocation signature can be visually detected from the interaction matrices for missed pairs. In addition, the sharp boundaries at translocation breakpoints on the 1D coverage profile can only be found in our predicted translocated chromosomal pairs. Thus, we believe that there are some translocated chromosomal pairs that are simply not reflected within Hi-C data, or the validation data may be incorrect, e.g., due to the variation among the K562 lines. We further examined four more cancer cell lines, including HelaS3 (cervical carcinoma), LNCaP, PANC-1, and T47D (breast cancer), for which FISH data were available for validation. We found that the rank product and the maximum interaction perform better than the Gini index in LNCaP, T47D, and PANC-1, whereas the rank product and Gini index are more predictive in HelaS3 (Additional file 1: Fig. S12E).

### HiNT detects translocation breakpoints at single base-pair resolution using Hi-C chimeric reads

Once a chromosomal pair containing a translocation is identified based on the rank product, HiNT searches for the translocation breakpoint. For a translocation, the 1D row/column-sum profile should change abruptly at the breakpoint (Additional file 1: Fig. S13, and Additional file 1: Fig. S15A). To identify this point, we use a change point detection method called *breakpoints* from the R package *strucchange* [31], which adopts a linear model to detect one or several change points in multivariate time series*.* However, the majority of the change points detected by *breakpoints* are the result of lower mappability and unremoved compartment effects and thus should not be identified as the translocation breakpoints (Additional file 1: Fig. S15A). To remove these false positives, we impose a filtering step in which only those with one quadrant (unbalanced translocation) or two diagonally opposite quadrants (balanced translocation) around the candidate breakpoint have very high interactions (Additional file 1: Fig. S15, “Methods”). Here, we define a high interaction frequency as being greater than the 99th percentile of all the interactions between the two chromosomes.

Next, we determine the precise coordinates of the breakpoints by using *ambiguous* chimeric reads [16] (Fig. 1a). These reads have their primary alignment near a breakpoint in one chromosome (e.g., chrA) and their clipped part align near a breakpoint in another chromosome (e.g., chrB). HiNT provides the intervals in which the breakpoints occur (100 kb resolution) and, as long as the breakpoint does not occur in an unmappable region, the exact location of the breakpoint (1 bp resolution).

### Hi-C can supplement WGS by locating translocation breakpoints in repetitive regions

**Fig. 5:**
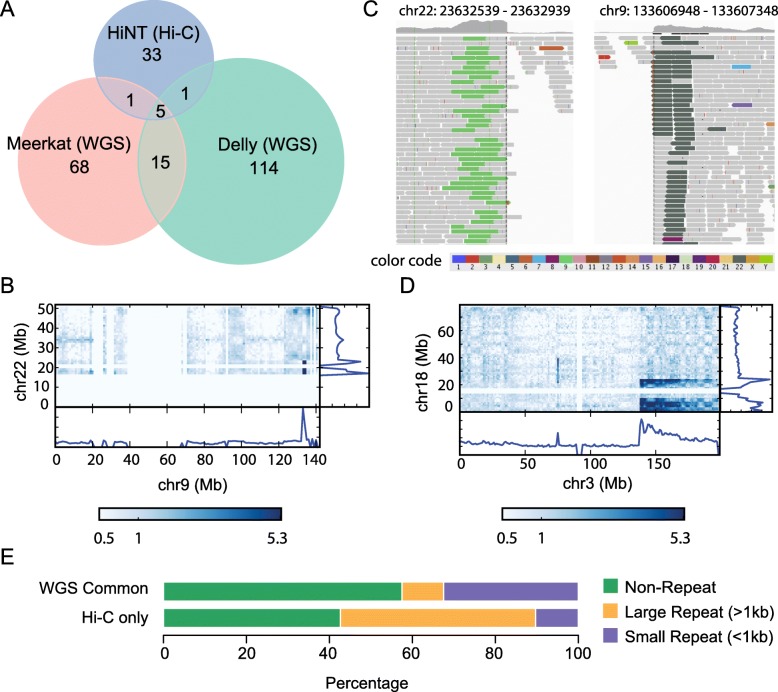
Comparison of breakpoints detected from Hi-C and WGS. **a** Overlap of the translocation breakpoints detected by Meerkat (WGS), Delly (WGS), and HiNT (Hi-C). **b** The Hi-C interaction map containing a breakpoint detected in both Hi-C and WGS. **c** The same exact breakpoint in panel B is captured in WGS. Discordant reads in light green (dark green) are paired end reads whose mates are found on chr9 (chr22). **d** Hi-C interaction map illustrating a clear case of a translocation detected only by HiNT. **e** Breakpoints detected in both Meerkat and Delly (“WGS Common”) and only in Hi-C only are classified into small repeat, large repeat, and non-repeat regions, showing that Hi-C is enriched for SVs involving large repeats

To assess its performance, we compare the translocation breakpoints determined from Hi-C using HiNT with those detected from WGS using Delly [32] and Meerkat [33]. In K562, 89 and 135 interchromosomal translocations are detected by Meerkat and Delly (see “Methods”), respectively, with only 20 translocations detected by both (Fig. 5a, Additional file 3: Table S2). This level of discrepancy is not unexpected [34] and is indicative of the difficulty of detecting SVs in general. When we intersect these 20 consensus WGS-based translocations with those detected by HiNT, we find that 5 are in common (Fig. 5a). Two additional ones were found by HiNT and either Meerkat or Delly but not both. In these 7 cases, the breakpoints were exactly the same at the nucleotide level, confirming the accuracy of the calls (Additional file 4: Table S3). An example is a translocation between chromosome 9 and 22 shown in Fig. 5b, with more than 100 supporting clipped reads in Hi-C data and many discordant reads in WGS data (Fig. 5c).

Thirty-three translocations are detected only from Hi-C data (Fig. 5a; listed in Additional file 5: Table S4). For example, a significant rank product score is found between chr3 and chr18 in the Hi-C interaction matrix (Fig. 5d), and three breakpoint regions are detected by HiNT including one validated by FISH [29] (Additional file 6: Table S5). However, few discordant reads are identified from WGS. A major reason for this difference is the low mappability around those breakpoints. As illustrated in Supplementary Figure 1, the long physical distance between Hi-C read pairs allow identification of translocations whose breakpoints occur in a repetitive region—the paired reads can “jump over” the repeat region and map to surrounding mappable regions, even though the breakpoint itself cannot be mapped. Indeed, we find that large repeat (> 1 kb) regions (as found in repBase [35]) make up a disproportionately large fraction of regions containing Hi-C-only breakpoints compared to WGS consensus breakpoints (Fig. 5e). We note that repetitive regions with high sequence divergence are mappable, but we used the term “repetitive region” for conceptual clarity.

For the translocations detected only in WGS, 6 out of 15 are missed in Hi-C simply because of the lower spatial resolution in Hi-C. Due to the nature of the assay, the coverage in the intergenic regions is especially sparse, regardless of the sequencing depth. As illustrated by two examples in Additional file 1: Fig. S16, when there is a translocation that turns out to be an insertion of a small segment from another chromosome, the Hi-C map does not show clear evidence (position indicated by a red dotted cross in the lower-left panel). When one zooms into that area, some interaction indicative of a translocated boundary is present; however, the interaction is too weak to be detected unless one lowers the detection criteria as to incur too many false positives. Two of the other nine cases appear to be complex SVs. In the two examples we show in Additional file 1: Fig. S17, discordant reads around the breakpoint are from two different chromosomes (indicated by different colors). Regardless of the exact details of the SV, it is clear that the Hi-C map (lower-left panel) does not capture the interactions; thus, HiNT cannot detect them. For the remaining seven cases, we believe these are false positive calls in WGS, often occurring in repetitive regions. We find that the discordant reads from WGS for these cases contain a large fraction of single nucleotide variants or have low mapping qualities, indicating issues in read alignment (Additional file 1: Fig. S18). Consistent with those being false positive WGS calls, no translocation-associated features are found in the Hi-C interaction maps. These analyses suggest that Hi-C is a powerful tool to detect translocations and can complement WGS, especially for detecting those with breakpoints in repetitive regions.

### HiNT outperforms existing tools on detecting translocations

Others have attempted to identify structural variants from Hi-C data. One approach is simply to visually inspect the interaction heatmaps—a low-resolution detection of breakpoints with poor scalability and reproducibility [28]. Better approaches search for regions that contain abnormal interaction frequencies based on normalized Hi-C interaction maps [6, 36]. However, such methods utilizing only contact frequencies cannot easily distinguish translocations from chromatin interactions, thus giving a high false discovery rate (FDR). A recent algorithm HiCtrans [23] identifies translocation breakpoints based on change point statistics obtained by scanning the interchromosomal contact maps of each chromosomal pair. However, searching the breakpoints across all interchromosomal contact maps leads to a high computational cost. For a comprehensive set of inter- and intrachromosomal translocations, one could integrate WGS, Hi-C, and optical mapping data [29]. However, in most cases, it is impractical to generate all these data types for a given sample. The method they used for Hi-C data [29] is hic_breakfinder, an iterative approach to locate local clusters that deviate from the expected interaction frequencies in a Hi-C contact matrix.

To compare the performance of these algorithms, we first apply HiCtrans [23] and HiNT to simulation data. Hic_breakfinder [29] is not used here because it requires the aligned reads in BAM format, but our simulation is matrix-based. Of the 21 simulated interchromosomal translocations (mix of balanced/unbalanced and heterozygous/homozygous translocations), HiNT identified 20 correctly while calling an additional 5 breakpoints (Additional file 1: Fig. S19A). The one missing translocation was located at the centromere of chr21 (Additional file 1: Fig. S19B). In contrast, HiCtrans called 531 translocations (distributed across 100 different chromosomal pairs), but none were bona fide translocations (Additional file 1: Fig. S19C).

We also compared HiNT, HiCtrans [23], and hic_breakfinder [29] on the K562, LNCaP, PANC-1, and T47D data. As shown in Additional file 1: Fig. S19D-E, HiNT has the highest AUC measure in most cell lines (0.85 vs 0.78 and 0.77 in K562, 0.98 vs 0.96 and 0.93 in LNCaP, 0.84 vs 0.87 and 0.7 in PANC-1 and 0.97 vs 0.84 and 0.93 in T47D, see “Methods”) as well as the best precision-recall curve. Additionally, in K562, we found that while HiCtrans identified 132 translocated chromosomal pairs, which is more than half of the number of all chromosomal pairs, only 10 of them contain known translocations. Among all 931 breakpoints (~ 1 Mb resolution) identified by HiCtrans, only 2 of them cover what are detected from WGS by both Meerkat and Delly (Additional file 1: Fig. S19F). On the other hand, hic_breakfinder identified 77 breakpoints (~ 100 kb resolution). Among these breakpoints, 4 are identified by both Meerkat and Delly (Additional file 1: Fig. S19F). This suggests a higher false discovery rate of HiCtrans and hic_breakfinder than HiNT. Furthermore, we found that 60% (24/40) of HiNT-identified breakpoints can also be identified by other methods. In contrast, this value is only 35% (26/77) and 3.0% (27/931) for breakpoints output from hic_breakfinder and hictrans, respectively (Additional file 1: Fig. S19F). Collectively, HiNT-TL outperforms HiCtrans and hic_breakfinder in both specificity and accuracy.

## Conclusion

Robust identification of SVs remains paramount to accurate inference of long-range interactions from Hi-C data. We have shown that HiNT can be used to identify CNVs and interchromosomal translocations with split read support for breakpoints whenever possible, and that it outperforms existing methods. We found that the other methods give inconsistent performance, depending on the extent of genomic alterations in the sample; this may be partially due to the less robust scheme for parameter tuning or the use of the hidden Markov Model [37]. Although not as sensitive as WGS data in general, Hi-C data can be surprisingly effective for CNV and translocation detection despite its less even coverage, and it can complement WGS data for detection of translocations in repetitive regions. As new technologies for capturing three-dimensional interactions are introduced, further computational methods will be needed to avoid the confounding effects of SVs.

## Methods

### Data sources

Hi-C data: in-situ Hi-C data in cancer cell line K562 and in normal cell lines including GM12878, HMEC, HUVEC, IMR90, and NHEK were obtained from GEO (Gene Expression Omnibus) with the accession number GSE63525 [16]. All the normal cell line data were combined to create the background Hi-C interaction matrix. Hi-C data for HelaS3, LNCaP, PANC-1, Caki2, and T47D, which were generated by the Dekker lab [38], were downloaded from the ENCODE website. Hi-C data in MCF7 and CHM13 were downloaded from GEO (GSM1631185) and the Telomere-to-Telomere consortium [39], respectively (see details in Additional file 2: Table S1).

WGS data: We downloaded the BAM file for NA12878 WGS data from the 1000 genomes project [40], and the BAM file for K562 WGS data from the GDC legacy archive of the Cancer Cell Line Encyclopedia (CCLE) project [41]. Raw FASTQ files in CHM13, LNCaP, and MCF7 were downloaded from SRA (Sequence Read Archive; see details in Additional file 2: Table S1), and FASTQ files in PANC-1 and Caki2 were obtained from a previous publication [29].

### CNV identification from WGS

BIC-seq2 [20] was used to derive CNV segments from WGS read coverage data. For the segmentation step, we used *binsize* = 50,000 bp and *λ* = 50 to determine the final CNV breakpoints in NA12878. *λ* is a parameter that controls the smoothness (the number of breakpoints) of the final CNV profile. chrY and chrM were excluded from the analysis.

### Definition of copy ratios in Hi-C and WGS data

Copy ratio is defined as the ratio of observed and expected values. In Hi-C, observed values are the residuals from GAM Poisson regression, and expected values are set to zero. In WGS, observed values are read coverage, and expected values are estimated by a semi-parametric regression model via BIC-seq2 [20].

### Simulation of interchromosomal translocations in Hi-C contact maps

The simulation pipeline defines two random coordinates from distinct chromosomes as the origin and destination of the translocation (e.g., x on chr1, and y on chr2). Then, it creates the translocated version of interaction matrices for chr1 to chr1, chr2 to chr2, and chr1 to chr2 via rearranging the original interaction probabilities.

### SV detection from WGS

SV detection from WGS was carried out using Delly and Meerkat. Default parameters were used to run Delly. Only translocations that passed the internal quality control and were marked as “PRECISE” in Delly were used for comparison. Default parameters were used to run Meerkat, and filtering was performed according to the post-processing steps described in the tool manual. Only valid precise interchromosomal translocations were kept for comparison. Translocation breakpoints located at pseudo-chromosomes are removed in both Meerkat and Delly for the comparison.

### Gini index calculation

For each Hi-C interchromosomal interaction matrix M (at 1 Mb resolution), we first sorted the contact regions, based on the adjusted contact frequencies between these two regions, from lowest to highest, then calculated the cumulated contact frequencies of matrix M. Regions that did not form contacts with any other regions were excluded. A plot of this functional relationship is called a Lorenz curve. The Gini index is computed as twice the area between the Lorenz curve and the diagonal.

### Breakpoint filtering

To remove false discovered change points, we first construct two-dimensional Cartesian coordinate systems originating from the intersection of each pair of candidate breakpoints. For each coordinate system, we then define four, 5-bin-by-5-bin quadrants around the origin, and we calculate the average interaction frequency within each quadrant (Additional file 1: Fig. S15A). The valid breakpoints for translocations should have only one (unbalanced translocation) or two (balanced translocation) quadrants with very high interactions, and the remaining quadrants should have lower interaction frequencies (Additional file 1: Fig. S15B upper panel). More specifically, for balanced translocations, the two quadrants with high interaction frequencies should diagonally oppose each other (Additional file 1: Fig. S15B upper panel). If zero, three, or all quadrants have high interaction frequencies, the proposed breakpoints are considered false positives and removed (Additional file 1: Fig. S15B lower panel). Here, we define a high interaction frequency as being greater than the 99th percentile of all the interactions between the two chromosomes.

### ROC curves of HiCtrans and HiC_breakfinder on translocated chromosomal pair prediction

To create ROC curves for the evaluation of translocated chromosomal pair prediction, we rank all the chromosomal pairs first. Both HiCtrans and hic_breakfinder output a score (entropy ratio in HiCtrans, and log-odds in hic_breakfinder) to measure the strength of each breakpoint call. We assign each chromosomal pair a representative score by taking the score of the most significant breakpoint that is located in this chromosomal pair. The chromosomal pairs are then ranked by the representative scores. ROC curves and AUC values are calculated by using the R package *ROCR* [42]; *p* values of the statistical test used to compare ROC curves were calculated by the R package *pROC* [43]. The chromosomal pairs reported in the literature or validated by FISH experiments are used as true positives here.

### Details of the HiNT pipeline

1. HiNT-PRE: Raw Hi-C data in FASTQ format are aligned to a reference genome (hg19) via bwa-mem (version 0.7.17-r1188): bwa mem -SP5M bwaIndex/hg19.fa in1.fq in2.fq. Read pairs that are both uniquely mapped to the genome are collected as valid pairs. However, 10–20% of the remaining Hi-C read pairs contain at least one chimeric read with split alignments. Chimeric pairs with one read uniquely mapped and the other chimeric, due to ligation, are defined as *unambiguous* chimeras [16] and counted as valid pairs. All other chimeric pairs are classified as *ambiguous* [16] chimeras and are used to identify translocation breakpoints at single base-pair resolution. All the unmapped, multi-mapped, and PCR duplicated read pairs are discarded from our analysis. All pairs are classified by pairtools (https://github.com/mirnylab/pairtools). Then, a Hi-C interaction matrix is generated from all the valid pairs by cooler [27] or juicer tools [44] at 50 kb, 100 kb, 1 Mb, or at a user-specified resolution.

2. HiNT-CNV: First, a 1D coverage profile for each 50-kb bin (default) is calculated along the whole genome using an unnormalized contact matrix. Bin size can be specified by users based on the sequencing depth and accuracy need. Then, a GAM regression with a Poisson link function is performed to remove the known Hi-C biases with pre-calculated GC content, mappability, and the number of restriction sites in each bin. In this study, we used the ENCODE 50mer mappability track downloaded from the UCSC table browser (https://hgdownload.soe.ucsc.edu/goldenPath/hg19/encodeDCC/wgEncodeMapability/wgEncodeCrgMapabilityAlign50mer.bigWig). As the local alignment strategy used in BWA-MEM may result in alignments of different lengths, using the mappability track of smaller fragment length, rather than the fixed 50mer track, may be more conservative. If desired, users can choose (via --maptrack) 24mer or 36mer tracks, also available from the UCSC table browser. Then, the segmentation method of BIC-seq is applied to the regression residuals to identify the breakpoints and generate the final CNV profile.

3. HiNT-TL: Translocation detection is performed in three steps; determination of the translocated chromosomal pairs, identification of the rough breakpoint regions, and determination of the exact breakpoints at single base pair resolution. To determine the translocated chromosomal pairs, 1-Mb-binned and genome-wide normalized interchromosomal interaction matrices are taken as input. To remove the effects of A/B compartments, a background model is created by averaging multiple in situ Hi-C data in normal cell lines (Additional file 2: Table S1). Our background model is built from five normal cell types. As these five are unlikely to be representative of all cell types, users may use their own cell type-specific background matrix or build average matrices by leveraging data from other tissues. Each interchromosomal interaction matrix is corrected with the background by taking the ratio between the original signals and the background signals. Then, for each possible chromosomal pair, Gini index and the maximum contact frequency are calculated. Then, a rank product score is computed *RP*_*i*_ = (*R*_gini,*i*_/*n*) ∗ (*R*_*mif*,*i*_/*n*), where *R*_gini,*i*_ and *R*_*mif*,*i*_ are the ranks of matrix *i* based on Gini index and maximum interaction frequency, respectively, and *n* is the total number of interchromosomal interaction matrices. Chromosomal pairs with *RP*_*i*_ ≤ 0.05 are defined as the potential translocated chromosomal pairs.

HiNT then calculates the 1D coverage profiles by calculating the sum of each row and column of the adjusted interchromosomal interaction matrices for those predicted translocated chromosomal pairs. It then applies the function *breakpoint* in the R package *strucchange*, a function with high computing performance that allows simultaneous estimation of multiple breakpoints in a given time series data, to the coverage profiles to identify all change points. The translocation rough breakpoint regions are further decided after the filtering step as we described in Additional file 1: Fig. S10.

To get the precise breakpoints at single base-pair resolution, HiNT uses the soft-clipped read-based algorithm that is commonly used for WGS SV prediction. Translocation breakpoints that are covered by at least one split read pair with one end mapped to the rough breakpoint region on one chromosome, and the other end mapped to the rough breakpoint region on another chromosome are reported at single base-pair resolution; otherwise, the predicted rough breakpoint regions will be reported. Not all the breakpoints are expected to have supported clipped reads due to the non-uniform distribution of read coverage in Hi-C data.

## Supplementary information

Additional file 1: **Figure S1.** Hi-C data is superior to WGS in variation detection in repetitive regions. A, Illustration of a fused chromosome with a breakpoint located in repetitive region. B-C, The distribution of the real distances (pink) between two mates, and the insert sizes (light blue) in WGS (B) and Hi-C (C). D, Reads can be correctly mapped to the reference genome if repeat size is less than the insert size in WGS. E, Reads cannot be correctly mapped to the reference genome if repeat size is larger than the insert size in WGS. F, Reads surrounding the repetitive regions can be used to detect the breakpoint in Hi-C. **Figure S2.** Overview of the HiNT workflow. HiNT has three components: HiNT-PRE, HiNT-CNV, and HiNT-TL. HiNT-PRE preprocesses Hi-C data to generate the contact matrix; HiNT-CNV performs CNV detection; and HiNT-TL detects translocation breakpoints at 100 kb as well as base-pair resolution. **Figure S3.** Correlation between the natural log of 1D coverage and the number of restriction sites (left), GC content (middle), and mappability (right) in each 50 kb bin in GM12878 (A) and K562 (B) cell. **Figure S4.** CNVs detected by HiNT from Hi-C are consistent with those detected from WGS. A-B, Correlation of log2 copy ratios in each bin (50 kb) detected from WGS and Hi-C (HiNT) in K562 (A) and GM12878 (B). C-D, Correlation of log2 copy ratios in each bin (50 kb) detected from WGS and Hi-C (HiCnv) in K562 (C) and GM12878 (D). E-F, Correlation of log2 copy ratios in each bin (50 kb) detected from WGS and Hi-C (OneD) in K562 (E) and GM12878 (F). **Figure S5.** Copy number inference in GM12878 cells. A, Comparison of log2 copy ratios calculated using regression residuals from Hi-C (blue) and using read coverage from WGS (orange). B, Comparison of CNV profiles from Hi-C and WGS after segmentation. Red, green and gray bars represent copy gain (log2 copy ratio > 0.3), copy loss (log2 copy ratio < − 0.3), and copy neutral regions (log2 copy ratio between − 0.3 and 0.3), respectively. C, Schematic of the consistency analysis. CNV segment detected from Hi-C is consistent with that detected from WGS if the overlapped region is larger than 50% of the original segment size, and vice versa. D, The number of CNV segments (categorized by size) detected from Hi-C that are also supported by WGS. Specifically, 92%, 28%, and 60% of the large CNV segments identified by HiNT, HiCnv, and OneD are supported by those from WGS, respectively. The overlap criteria for consistency are shown in panel C. **Figure S6.** Evaluation of copy number inference from Hi-C data in Caki2 cells. A, Correlation of log2 copy ratios in each bin (50 kb) detected from WGS and HiNT, HiCnv, and OneD in Caki2. B, Comparison of CNV profiles from Hi-C and WGS after segmentation. Red, green and gray bars represent copy gain (log2 copy ratio > 0.3), copy loss (log2 copy ratio < − 0.3), and copy neutral regions (log2 copy ratio between − 0.3 and 0.3), respectively. C, The number of CNV segments (categorized by size) detected from Hi-C that are also supported by WGS. Specifically, 84%, 76%, and 49% of the large CNV segments identified by HiNT, HiCnv, and OneD are supported by those from WGS, respectively. The overlap criteria for consistency are shown in Supp. Fig. 5c. **Figure S7.** Evaluation of copy number inference from Hi-C data in LNCaP cells. A, Correlation of log2 copy ratios in each bin (50 kb) detected from WGS and HiNT, HiCnv, and OneD in LNCaP. B, Comparison of CNV profiles from Hi-C and WGS after segmentation. Red, green and gray bars represent copy gain (log2 copy ratio > 0.3), copy loss (log2 copy ratio < − 0.3), and copy neutral regions (log2 copy ratio between − 0.3 and 0.3), respectively. C, The number of CNV segments (categorized by size) detected from Hi-C that are also supported by WGS. Specifically, 79%, 84%, and 54% of the large CNV segments identified by HiNT, HiCnv, and OneD are supported by those from WGS, respectively. The overlap criteria for consistency are shown in Supp. Fig. 5c. **Figure S8.** Evaluation of copy number inference from Hi-C data in MCF7 cells. A, Correlation of log2 copy ratios in each bin (50 kb) detected from WGS and HiNT, HiCnv, and OneD in MCF7. B, Comparison of CNV profiles from Hi-C and WGS after segmentation. Red, green and gray bars represent copy gain (log2 copy ratio > 0.3), copy loss (log2 copy ratio < − 0.3), and copy neutral regions (log2 copy ratio between − 0.3 and 0.3), respectively. C, The number of CNV segments (categorized by size) detected from Hi-C that are also supported by WGS. Specifically, 76%, 71%, and 49% of the large CNV segments identified by HiNT, HiCnv, and OneD are supported by those from WGS, respectively. The overlap criteria for consistency are shown in Supp. Fig. 5c. **Figure S9.** Evaluation of copy number inference from Hi-C data in PANC-1 cells. A, Correlation of log2 copy ratios in each bin (50 kb) detected from WGS and HiNT, HiCnv, and OneD in PANC-1. B, Comparison of CNV profiles from Hi-C and WGS after segmentation. Red, green and gray bars represent copy gain (log2 copy ratio > 0.3), copy loss (log2 copy ratio < − 0.3), and copy neutral regions (log2 copy ratio between − 0.3 and 0.3), respectively. C, The number of CNV segments (categorized by size) detected from Hi-C that are also supported by WGS. Specifically, 84%, 81%, and 58% of the large CNV segments identified by HiNT, HiCnv, and OneD are supported by those from WGS, respectively. The overlap criteria for consistency are shown in Supp. Fig. 5c. **Figure S10.** Evaluation of copy number inference from Hi-C data in CHM13 cells. A, Correlation of log2 copy ratios in each bin (50 kb) detected from WGS and HiNT, HiCnv, and OneD in CHM13. B, Comparison of CNV profiles from Hi-C and WGS after segmentation. Red, green and gray bars represent copy gain (log2 copy ratio > 0.3), copy loss (log2 copy ratio < − 0.3), and copy neutral regions (log2 copy ratio between − 0.3 and 0.3), respectively. C, The number of CNV segments (categorized by size) detected from Hi-C that are also supported by WGS. Specifically, 92%, 71%, and 95% of the large CNV segments identified by HiNT, HiCnv, and OneD are supported by those from WGS, respectively. The overlap criteria for consistency are shown in Supp. Fig. 5c. **Figure S11.** Rank Product approach accurately identifies simulated translocated chromosome pairs. Distribution of the maximum interaction frequency (left), the Gini Index in an interchromosome contact matrix (middle), and the rank product of these two (right) in Hi-C data with simulated translocations. **Figure S12.** Rank Product approach accurately identifies translocated chromosome pairs. A, The distribution of the maximum interaction frequency (MIF, left), the Gini Index (right), and the rank product of these two (Fig. 4a) in inter-chromosome contact matrices before (upper) and after (lower) adjustment in K652 cells. Chromosomal pairs in pink and blue correspond to two FISH-validated translocation pairs (chr1, chr21) and (chr1, chr18); the one in yellow corresponds to a chromosome pair (chr16, chr19) without translocation. B, AUROC values show either Gini Index or MIF perform better after the background subtraction in K562 cells. C-D, ROC curves (C) and precision-recall curves (D) of translocated chromosomal pairs predicted by using Gini Index only (orange), the maximum interaction only (dark green), and the rank product of these two (red) in K562 cells. E, Performance of rank product, Gini index, and the maximum interactions in HelaS3, LNCaP, Panc1, and T47D cells. **Figure S13.** Examples of chromosomal pairs with most significant rank product. A-D, Hi-C interchromosomal heatmaps and 1D coverage in original K562(left), background (middle), and adjusted K562 (K562/Background, right) data. Hi-C 1-D profiles (the sum of rows and columns of each inter-chromosomal interaction matrix) are shown along with the interaction maps. Translocation breakpoints are marked by red circles. **Figure S14.** Examples of missed translocated chromosomal pairs by HiNT. A-D, Hi-C interchromosomal heatmaps and 1D coverage in original K562(left), background (middle), and adjusted K562 (K562/Background, right) data. Hi-C 1-D profiles (the sum of rows and columns of each inter-chromosomal interaction matrix) are shown along with the interaction maps. **Figure S15.** Breakpoint detection and filtering. A, Candidate breakpoints (gray lines) detected by *strucchange* based on the 1D coverage profile (sum of rows and columns). Two-dimensional Cartesian coordinate systems originating from the intersection of each pair of candidate breakpoints are constructed; two examples are shown in the figure. B, Patterns of Hi-C interaction frequencies in four 5-bin-by-5-bin quadrants, that generated by the pair of breakpoints from both chromosomes. Valid translocation breakpoints are shown above the dash line, and invalid breakpoints are shown below. C, Translocation breakpoints (red dotted lines) after the filtering step. **Figure S16.** Examples of the small segment inter-chromosomal insertional translocation that is detected from only WGS. A, The distribution of discordant reads and clipped reads around the translocation breakpoints detected from WGS on chr1 and chr17 (upper); Hi-C interaction heatmap across the whole chromosomes (bottom left) and regions around breakpoints (bottom right). B, Similar to A, but the translocation between chr3 and chr10. In the IGV screenshot (WGS reads distribution), each color bar represents a SNV (single nucleotide variant), and the colored reads are paired end reads coded by the chromosome on which their mates can be found. The color code for discordant reads is shown at the bottom. **Figure S17.** Examples of the complex SVs. A, The distribution of discordant reads and clipped reads around the translocation breakpoints detected from WGS on chr3 and chr9 (upper); Hi-C interaction heatmap across the whole chromosomes (bottom left) and regions around breakpoints (bottom right). B, Similar to A, but the translocation between chr3 and chr12. In the IGV screenshot (WGS reads distribution), each color bar represents a SNV (single nucleotide variant), and the colored reads are paired end reads coded by the chromosome on which their mates can be found. The color code for discordant reads is shown at the bottom. **Figure S18.** Examples of the false positives that identified from WGS data. A, The distribution of discordant reads and clipped reads around the translocation breakpoints detected from WGS on chr17 and chr20 (upper); Hi-C interaction heatmap across the whole chromosomes (bottom left) and regions around breakpoints (bottom right). B, Similar to A, but the translocation between chr19 and chr20. In the IGV screenshot (WGS reads distribution), each color bar represents a SNV (single nucleotide variant), and the colored reads are paired end reads coded by the chromosome on which their mates can be found. The color code for discordant reads is shown at the bottom. **Figure S19.** HiNT outperforms existing methods on translocation breakpoints detection in both simulated and real Hi-C data. A, The overlap of translocation breakpoints detected by HiNT and simulated true set. B, Hi-C interaction heatmap for the breakpoint that was missed by HiNT, the sum of rows and columns are shown along the matrix. C, The overlap of translocation breakpoints detected by HiCtrans and simulated true set. D-E, Evaluation of the performance of HiNT (red curve), HiCtrans (navy curve), and hic_breakfinder (purple curve) on translocated chromosome pairs prediction in K562, LNCaP, PANC-1, and T47D cells by ROC curves (D) and precision-recall curves (E). *P-values* (see Methods) for the AUC comparison between HiNT and HiCtrans/OneD are labeled in the figures. F, Intersections of the translocation breakpoints detected by Meerkat and Delly from WGS, and HiNT, HiCtrans and hic_breakfinder from Hi-C.

Additional file 2: Table S1. Datasets used in this study.

Additional file 3: Table S2. Translocation breakpoints detected by both Meerkat and Delly.

Additional file 4: Table S3. Translocation breakpoints identified by both Hi-C and WGS.

Additional file 5: Table S5. Translocation breakpoints identified by HiNT in K562 cells.

Additional file 6: Table S5. Details of breakpoints between chr3 and chr18.

Additional file 7: Review history.

## Data Availability

HiNT is available as open source at https://github.com/parklab/HiNT under the MIT license [45]. The original source script for this manuscript is stored with the digital object identifier (DOI) at 10.5281/zenodo.3669319 [46].
