# Supplementary material for: HiNT: a computational method for detecting copy number variations and translocations from Hi-C data
Source: Genome Biol. 2020 Mar 23;21:73. doi: 10.1186/s13059-020-01986-5 (PMC7087379; doi:10.1186/s13059-020-01986-5)
Supplement: Supplementary file 1 — Figure S1. Hi-C data is superior to WGS in variation detection in repetitive regions. A, Illustration of a fused chromosome with a breakpoint located in repetitive region. B-C, The distribution of the real distances (pink) between two mates, and the insert sizes (light blue) in WGS (B) and Hi-C (C). D, Reads can be correctly mapped to the reference genome if repeat size is less than the insert size in WGS. E, Reads cannot be correctly mapped to the reference genome if repeat size is larger than the insert size in WGS. F, Reads surrounding the repetitive regions can be used to detect the breakpoint in Hi-C. Figure S2. Overview of the HiNT workflow. HiNT has three components: HiNT-PRE, HiNT-CNV, and HiNT-TL. HiNT-PRE preprocesses Hi-C data to generate the contact matrix; HiNT-CNV performs CNV detection; and HiNT-TL detects translocation breakpoints at 100 kb as well as base-pair resolution. Figure S3. Correlation between the natural log of 1D coverage and the number of restriction sites (left), GC content (middle), and mappability (right) in each 50 kb bin in GM12878 (A) and K562 (B) cell. Figure S4. CNVs detected by HiNT from Hi-C are consistent with those detected from WGS. A-B, Correlation of log2 copy ratios in each bin (50 kb) detected from WGS and Hi-C (HiNT) in K562 (A) and GM12878 (B). C-D, Correlation of log2 copy ratios in each bin (50 kb) detected from WGS and Hi-C (HiCnv) in K562 (C) and GM12878 (D). E-F, Correlation of log2 copy ratios in each bin (50 kb) detected from WGS and Hi-C (OneD) in K562 (E) and GM12878 (F). Figure S5. Copy number inference in GM12878 cells. A, Comparison of log2 copy ratios calculated using regression residuals from Hi-C (blue) and using read coverage from WGS (orange). B, Comparison of CNV profiles from Hi-C and WGS after segmentation. Red, green and gray bars represent copy gain (log2 copy ratio > 0.3), copy loss (log2 copy ratio < − 0.3), and copy neutral regions (log2 copy ratio between − 0.3 and 0.3), respectively. C, Schem [file 13059_2020_1986_MOESM1_ESM.pdf]

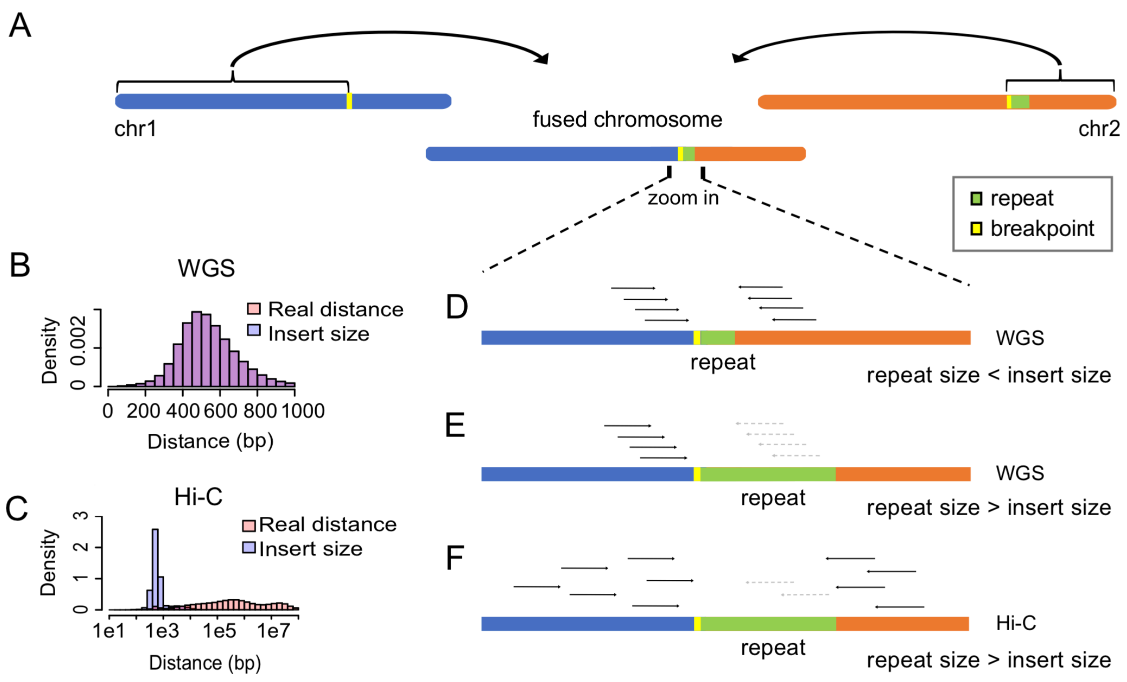

Fig. S1. Hi-C data is superior to WGS in variation detection in repetitive regions. A, Illustration of a fused chromosome with a breakpoint located in repetitive region. **B-C,** The distribution of the real distances (pink) between two mates, and the insert sizes (light blue) in WGS (B) and Hi-C (C). **D,** Reads can be correctly mapped to the reference genome if repeat size is less than the insert size in WGS. **E,** Reads cannot be correctly mapped to the reference genome if repeat size is larger than the insert size in WGS. **F,** Reads surrounding the repetitive regions can be used to detect the breakpoint in Hi-C.

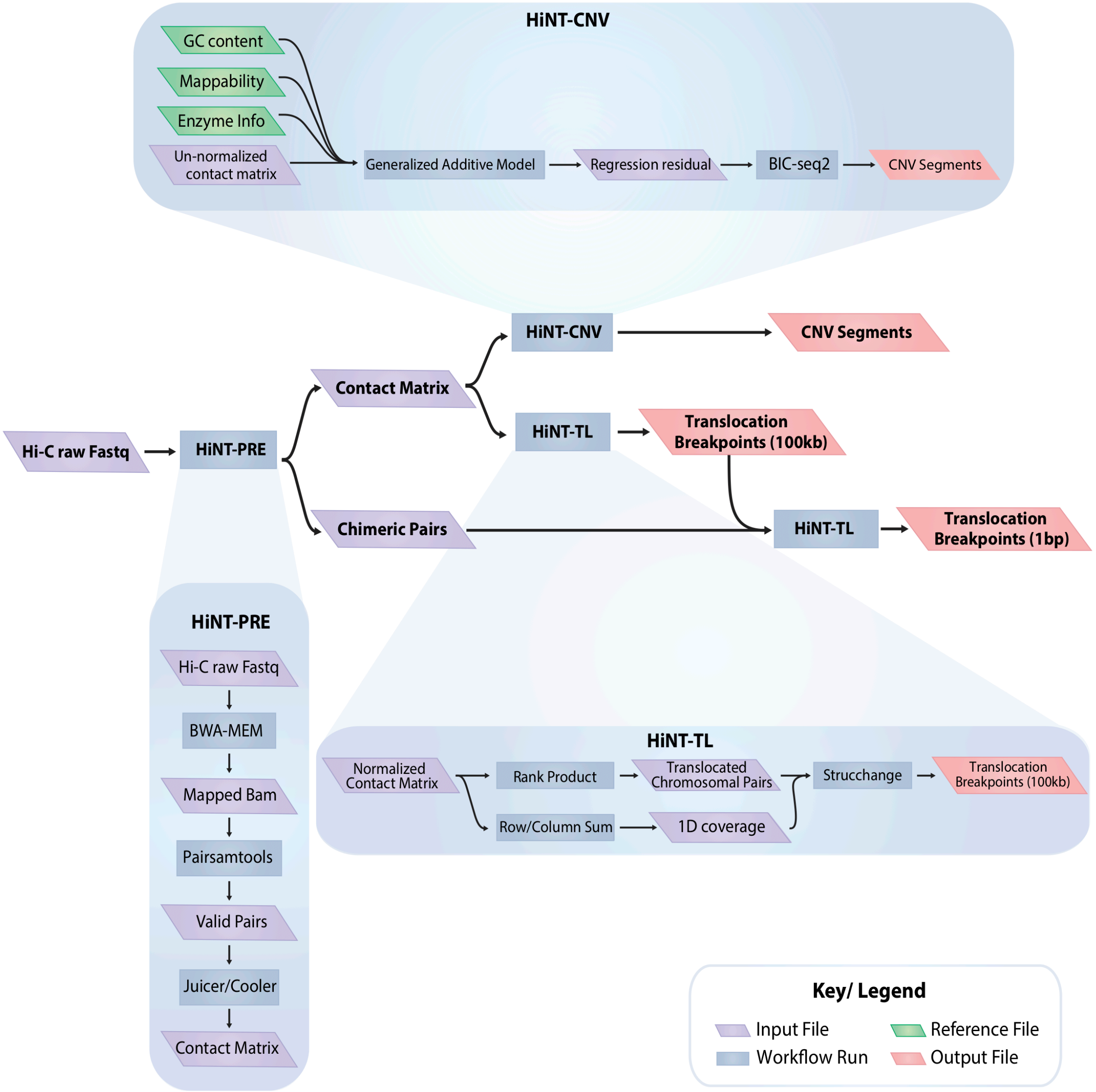

Fig. S2. Overview of the HiNT workflow. HiNT has three components: HiNT-PRE, HiNT-CNV, and HiNT-TL. HiNT-PRE preprocesses Hi-C data to generate the contact matrix; HiNT-CNV performs CNV detection; and HiNT-TL detects translocation breakpoints at 100kb as well as base-pair resolution.

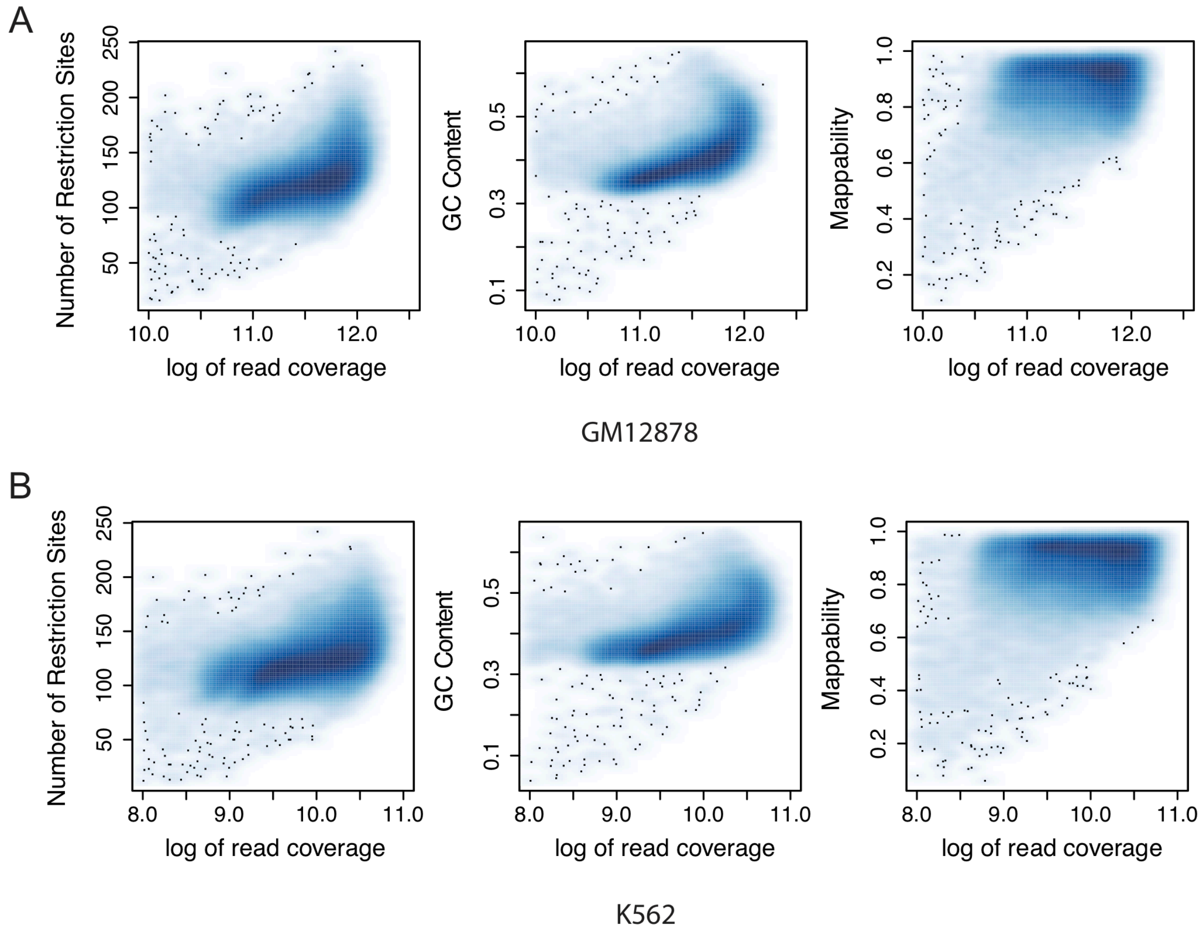

Fig. S3. **Correlation between the natural log of 1D coverage and the number of restriction sites** (left), **GC content** (middle), **and mappability** (right) in each 50kb bin in GM12878 (A) and K562 (B) cell.

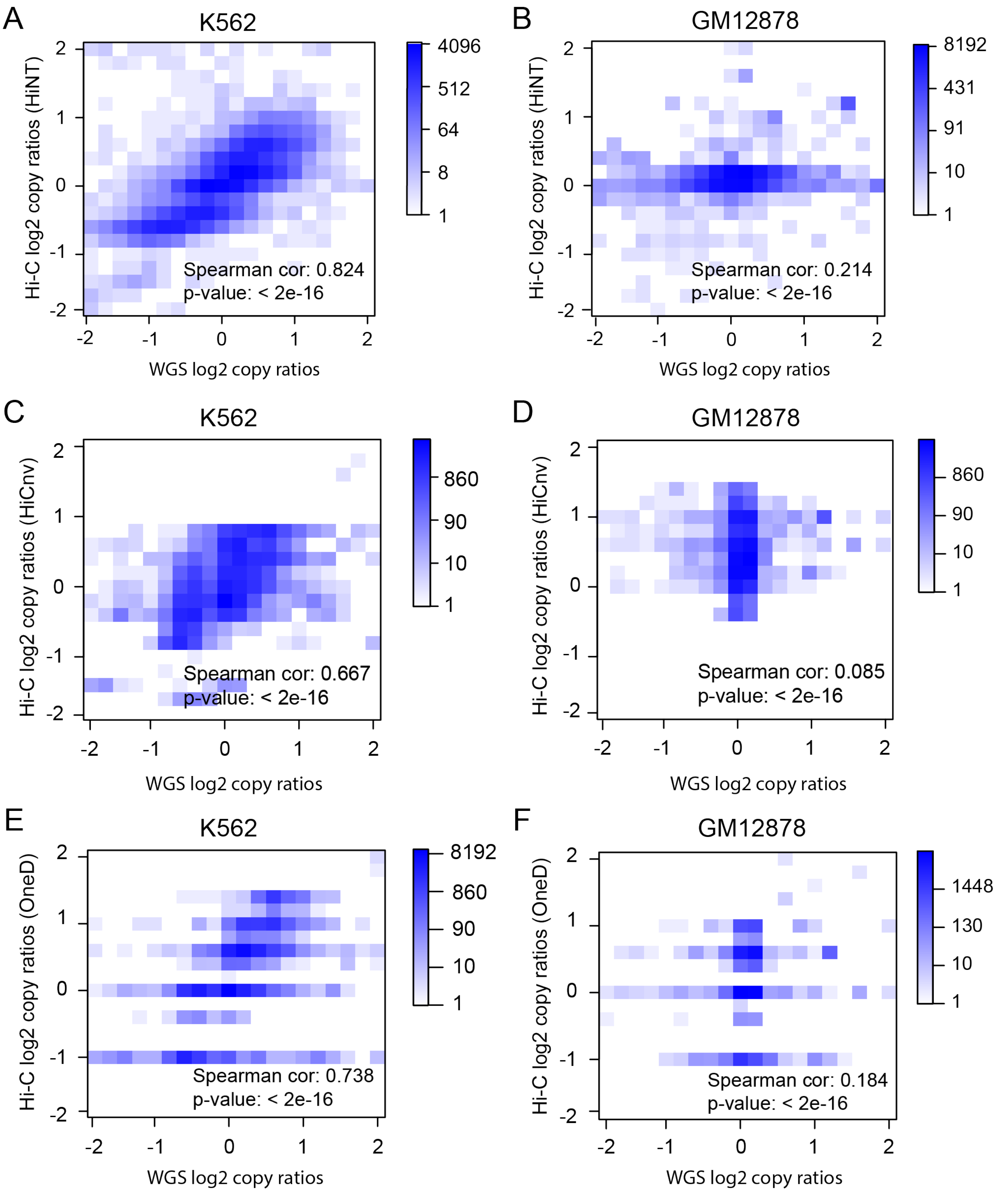

Fig. S4. **CNVs detected by HiNT from Hi-C are consistent with those detected from WGS. A-B,** Correlation of log2 copy ratios in each bin (50kb) detected from WGS and Hi-C (HiNT) in K562 (A) and GM12878 (B). **C-D,** Correlation of log2 copy ratios in each bin (50kb) detected from WGS and Hi-C (HiCnv) in K562 (C) and GM12878 (D). **E-F,** Correlation of log2 copy ratios in each bin (50kb) detected from WGS and Hi-C (OneD) in K562 (E) and GM12878 (F).

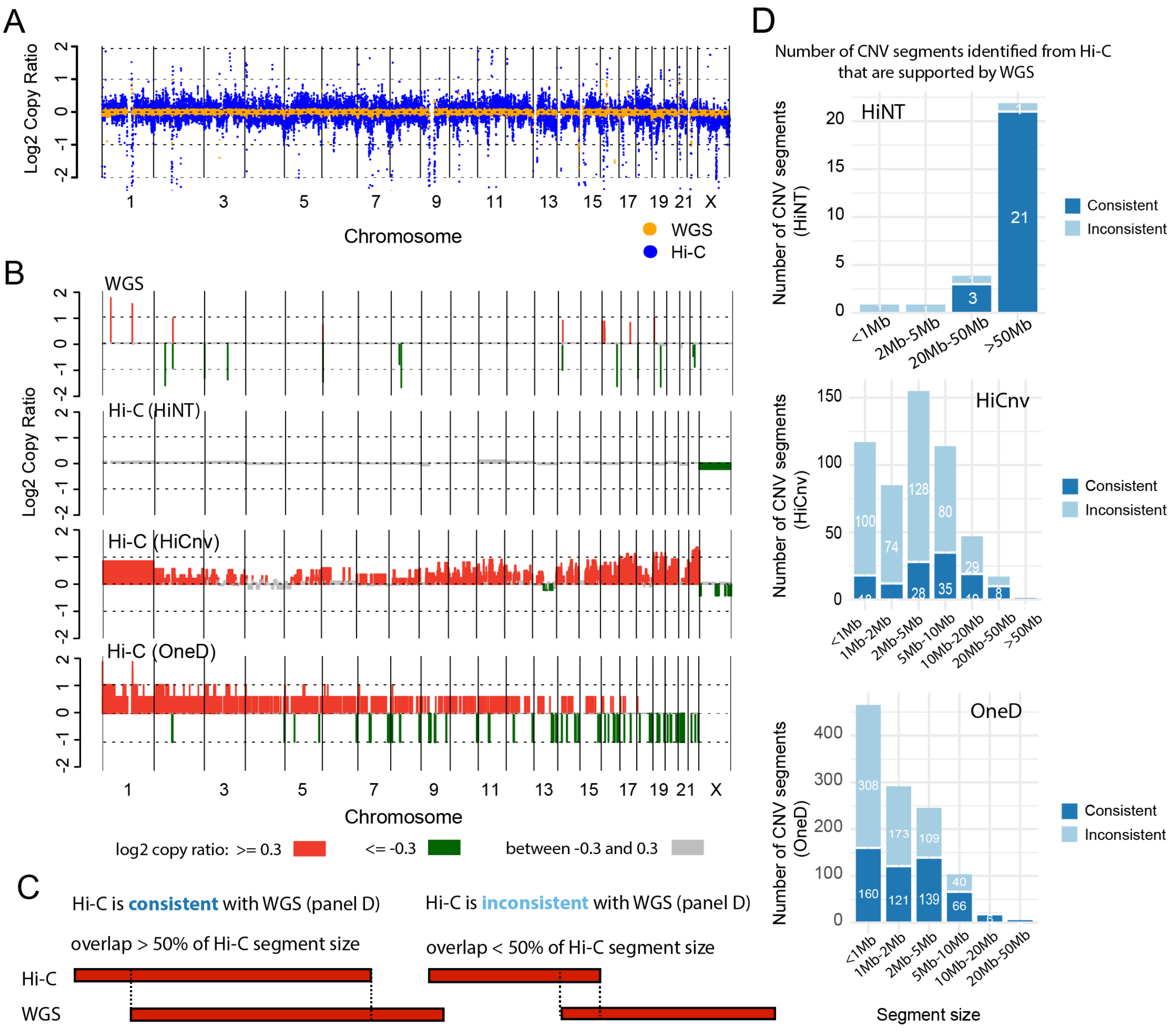

Fig. S5. **Copy number inference in GM12878 cells. A,** Comparison of log2 copy ratios calculated using regression residuals from Hi-C (blue) and using read coverage from WGS (orange). **B,** Comparison of CNV profiles from Hi-C and WGS after segmentation. Red, green and grey bars represent copy gain(log2 copy ratio > 0.3), copy loss(log2 copy ratio < -0.3), and copy neutral regions (log2 copy ratio between -0.3 and 0.3), respectively. **C,** Schematic of the consistency analysis. CNV segment detected from Hi-C is consistent with that detected from WGS if the overlapped region is larger than 50% of the original segment size, and vice versa. **D,** The number of CNV segments (categorized by size) detected from Hi-C that are also supported by WGS. Specifically, 92%, 28%, and 60% of the large CNV segments identified by HiNT, HiCnv, and OneD are supported by those from WGS, respectively. The overlap criteria for consistency are shown in panel C.

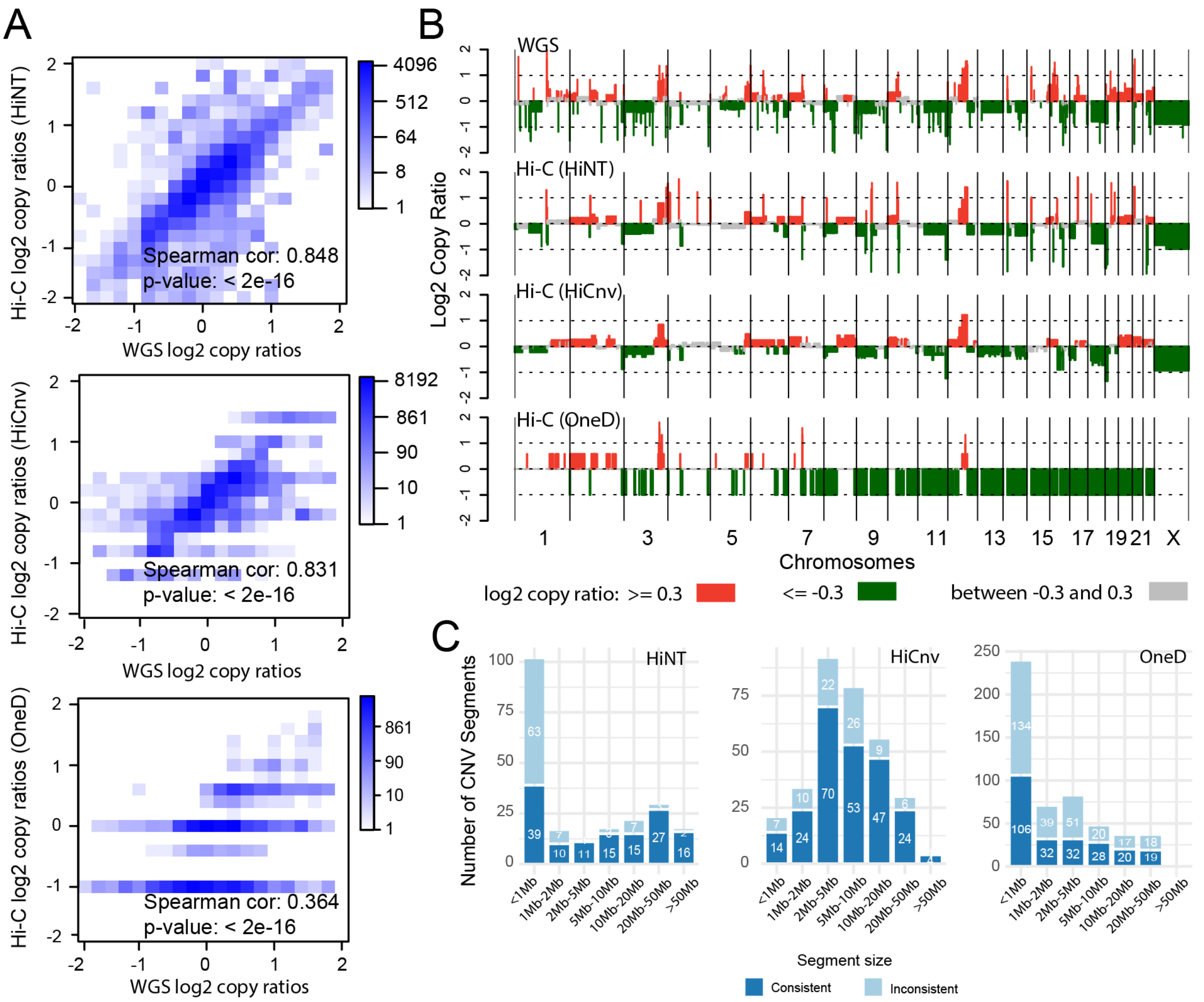

Fig. S6. **Evaluation of copy number inference from Hi-C data in Caki2 cells**. **A,** Correlation of log2 copy ratios in each bin (50kb) detected from WGS and HiNT, HiCnv, and OneD in Caki2. **B,** Comparison of CNV profiles from Hi-C and WGS after segmentation. Red, green and grey bars represent copy gain(log2 copy ratio > 0.3), copy loss(log2 copy ratio < -0.3), and copy neutral regions (log2 copy ratio between -0.3 and 0.3), respectively. **C,** The number of CNV segments (categorized by size) detected from Hi-C that are also supported by WGS.
Specifically, 84%, 76%, and 49% of the large CNV segments identified by HiNT, HiCnv, and OneD are supported by those from WGS, respectively. The overlap criteria for consistency are shown in Supp. Fig. 5C.

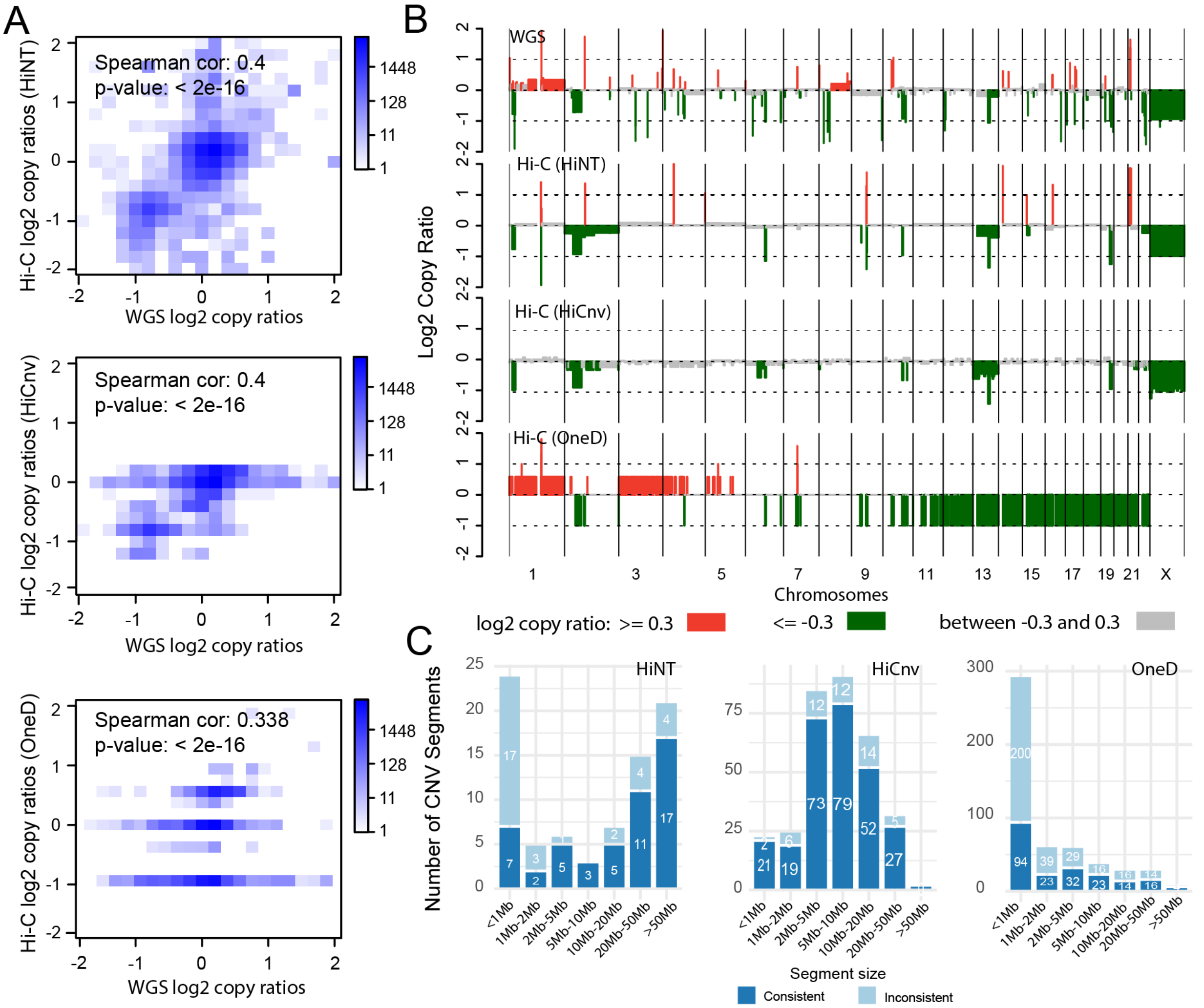

Fig. S7. **Evaluation of copy number inference from Hi-C data in LNCaP cells**. **A,** Correlation of log2 copy ratios in each bin (50kb) detected from WGS and HiNT, HiCnv, and OneD in LNCaP. **B,** Comparison of CNV profiles from Hi-C and WGS after segmentation. Red, green and grey bars represent copy gain(log2 copy ratio > 0.3), copy loss(log2 copy ratio < -0.3), and copy neutral regions (log2 copy ratio between -0.3 and 0.3), respectively. **C,** The number of CNV segments (categorized by size) detected from Hi-C that are also supported by WGS.
Specifically, 79%, 84%, and 54% of the large CNV segments identified by HiNT, HiCnv, and OneD are supported by those from WGS, respectively. The overlap criteria for consistency are shown in Supp. Fig. 5C.

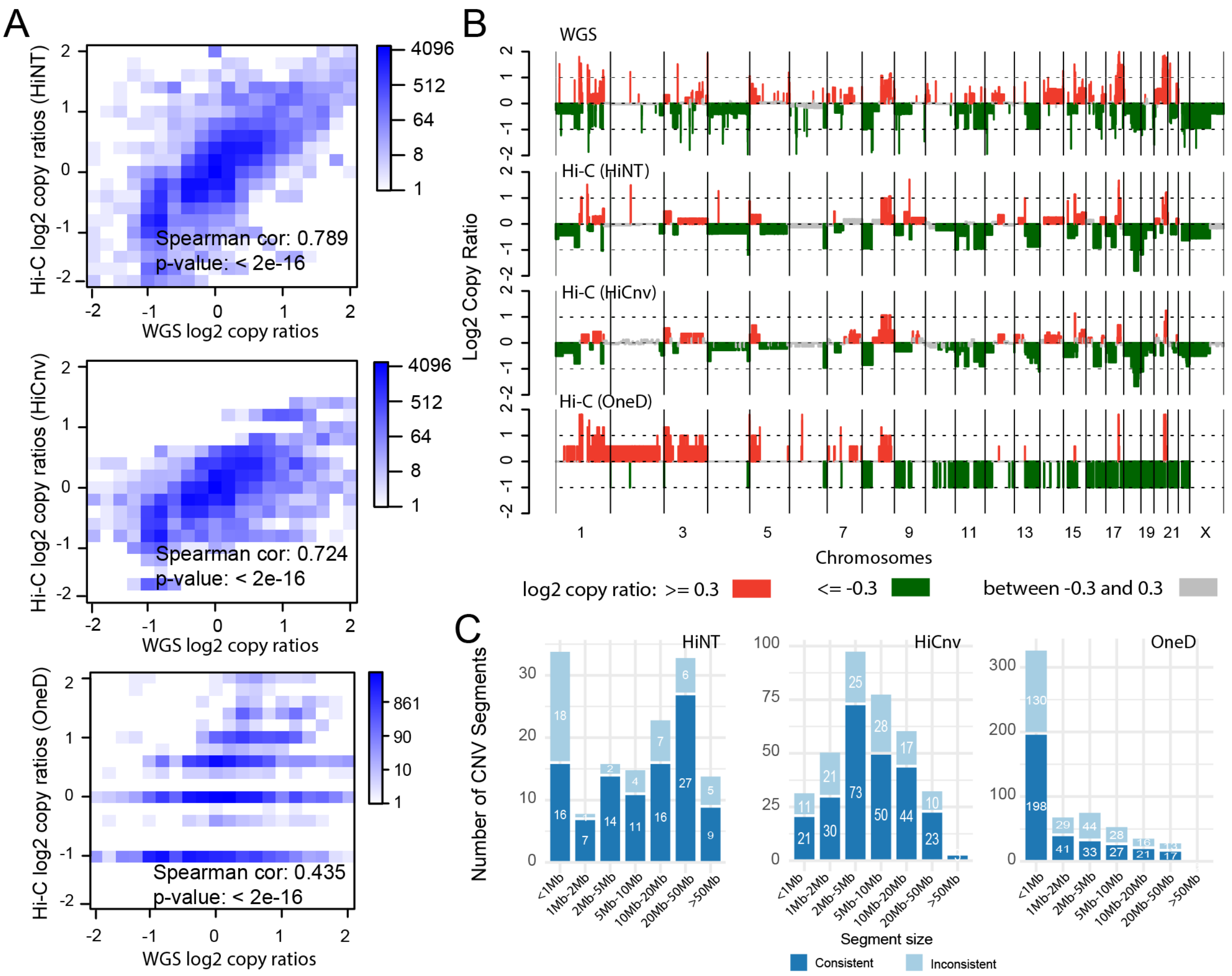

Fig. S8. **Evaluation of copy number inference from Hi-C data in MCF7 cells**. **A,** Correlation of log2 copy ratios in each bin (50kb) detected from WGS and HiNT, HiCnv, and OneD in MCF7. **B,** Comparison of CNV profiles from Hi-C and WGS after segmentation. Red, green and grey bars represent copy gain(log2 copy ratio > 0.3), copy loss(log2 copy ratio < -0.3), and copy neutral regions (log2 copy ratio between -0.3 and 0.3), respectively. **C,** The number of CNV segments (categorized by size) detected from Hi-C that are also supported by WGS.
Specifically, 76%, 71%, and 49% of the large CNV segments identified by HiNT, HiCnv, and OneD are supported by those from WGS, respectively. The overlap criteria for consistency are shown in Supp. Fig. 5C.

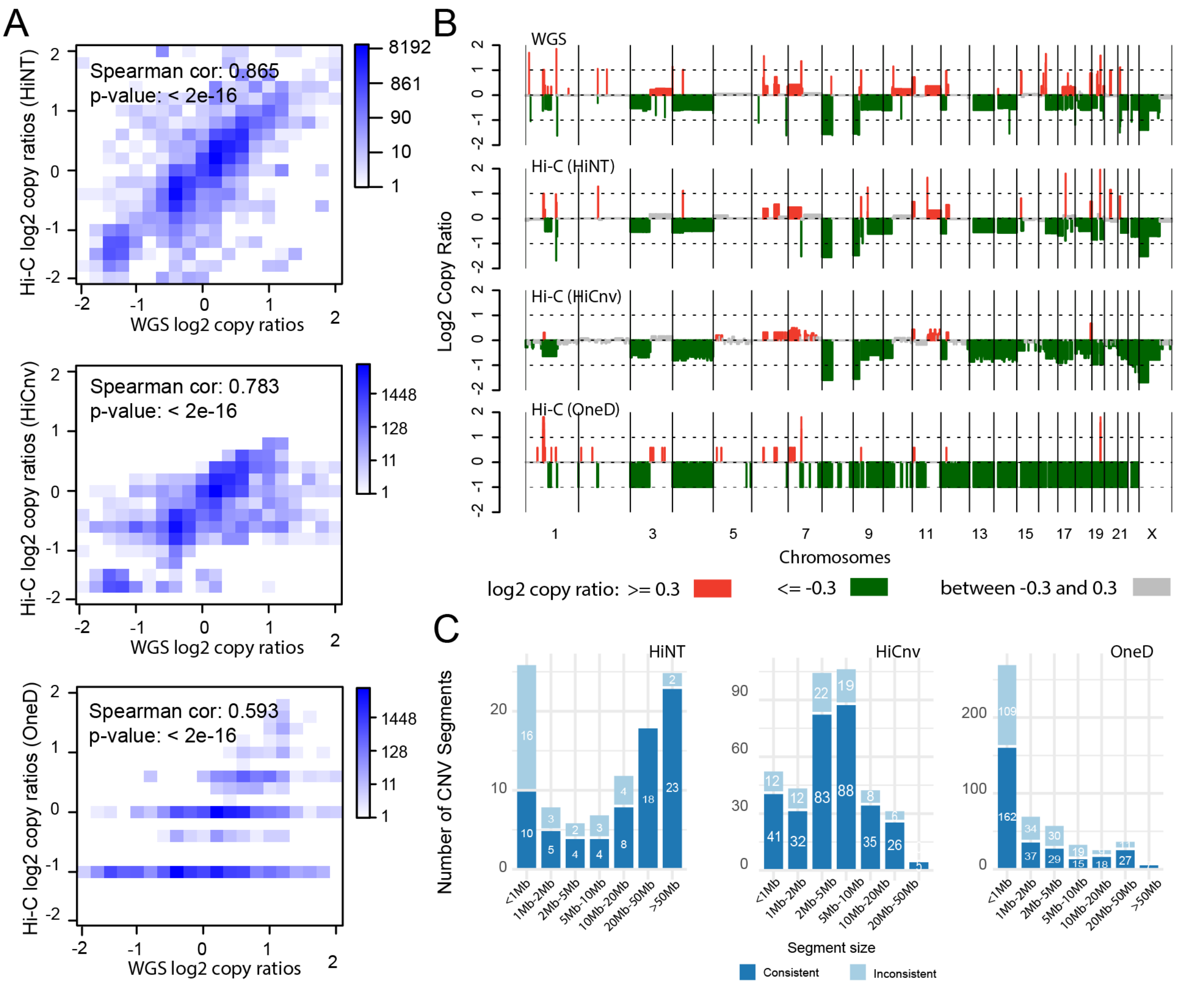

Fig. S9. **Evaluation of copy number inference from Hi-C data in PANC-1 cells. A,** Correlation of log2 copy ratios in each bin (50kb) detected from WGS and HiNT, HiCnv, and OneD in PANC-1. **B,** Comparison of CNV profiles from Hi-C and WGS after segmentation. Red, green and grey bars represent copy gain(log2 copy ratio > 0.3), copy loss(log2 copy ratio < -0.3), and copy neutral regions (log2 copy ratio between -0.3 and 0.3), respectively. **C,** The number of CNV segments (categorized by size) detected from Hi-C that are also supported by WGS.
Specifically, 84%, 81%, and 58% of the large CNV segments identified by HiNT, HiCnv, and OneD are supported by those from WGS, respectively. The overlap criteria for consistency are shown in Supp. Fig. 5C.

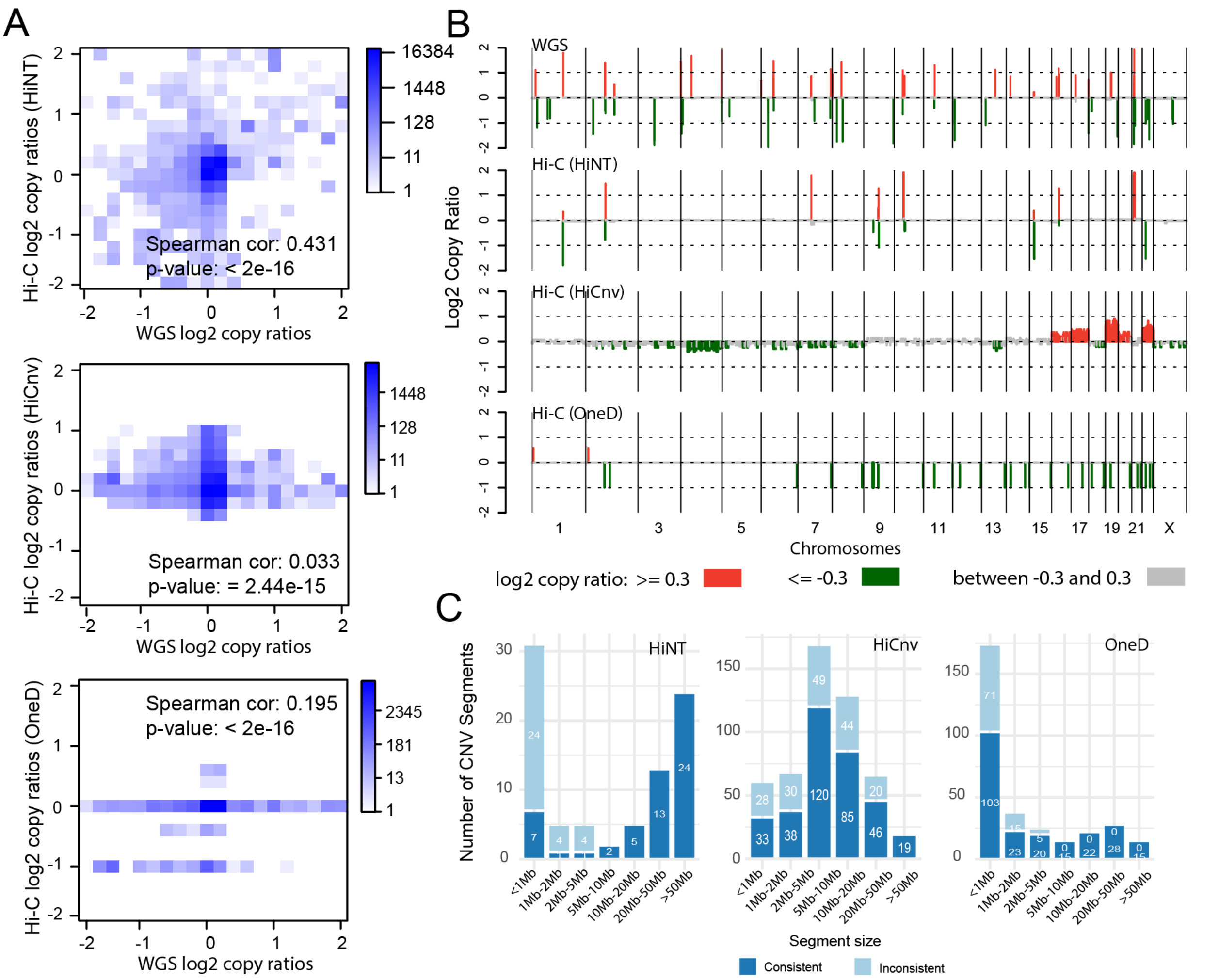

Fig. S10. **Evaluation of copy number inference from Hi-C data in CHM13 cells. A,** Correlation of log2 copy ratios in each bin (50kb) detected from WGS and HiNT, HiCnv, and OneD in CHM13. **B,** Comparison of CNV profiles from Hi-C and WGS after segmentation. Red, green and grey bars represent copy gain(log2 copy ratio > 0.3), copy loss(log2 copy ratio < -0.3), and copy neutral regions (log2 copy ratio between -0.3 and 0.3), respectively. **C,** The number of CNV segments (categorized by size) detected from Hi-C that are also supported by WGS.
Specifically, 92%, 71%, and 95% of the large CNV segments identified by HiNT, HiCnv, and OneD are supported by those from WGS, respectively. The overlap criteria for consistency are shown in Supp. Fig. 5C.

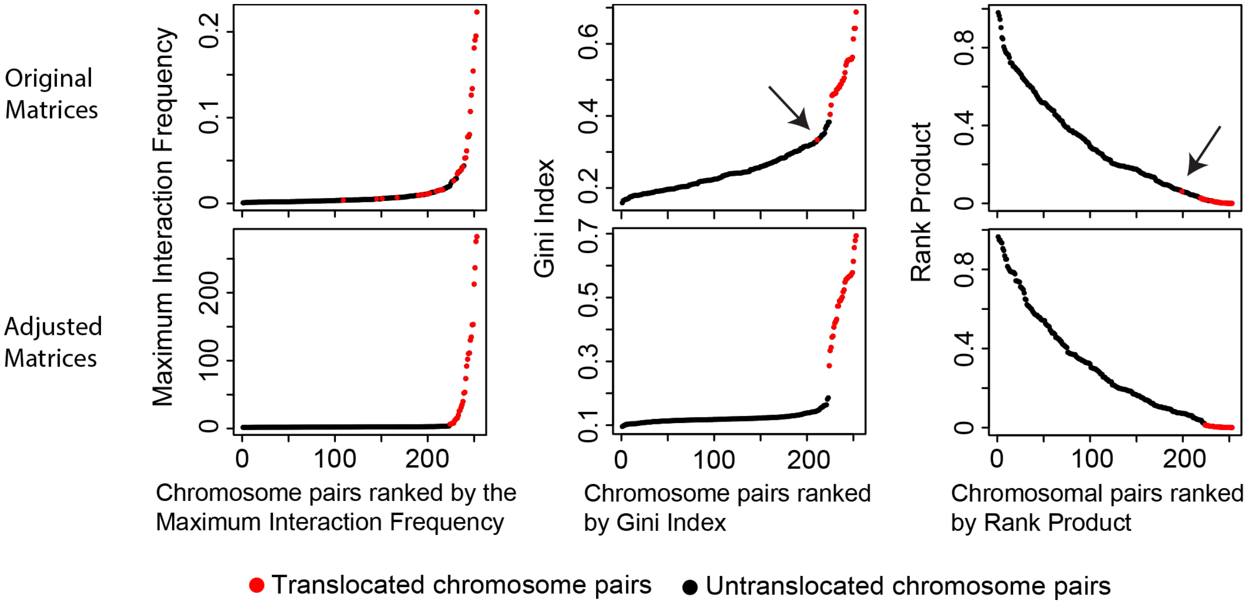

Fig. S11. Rank Product approach accurately identifies simulated translocated chromosome pairs. Distribution of the maximum interaction frequency (left), the Gini Index in an inter-chromosome contact matrix (middle), and the rank product of these two (right) in Hi-C data with simulated translocations.

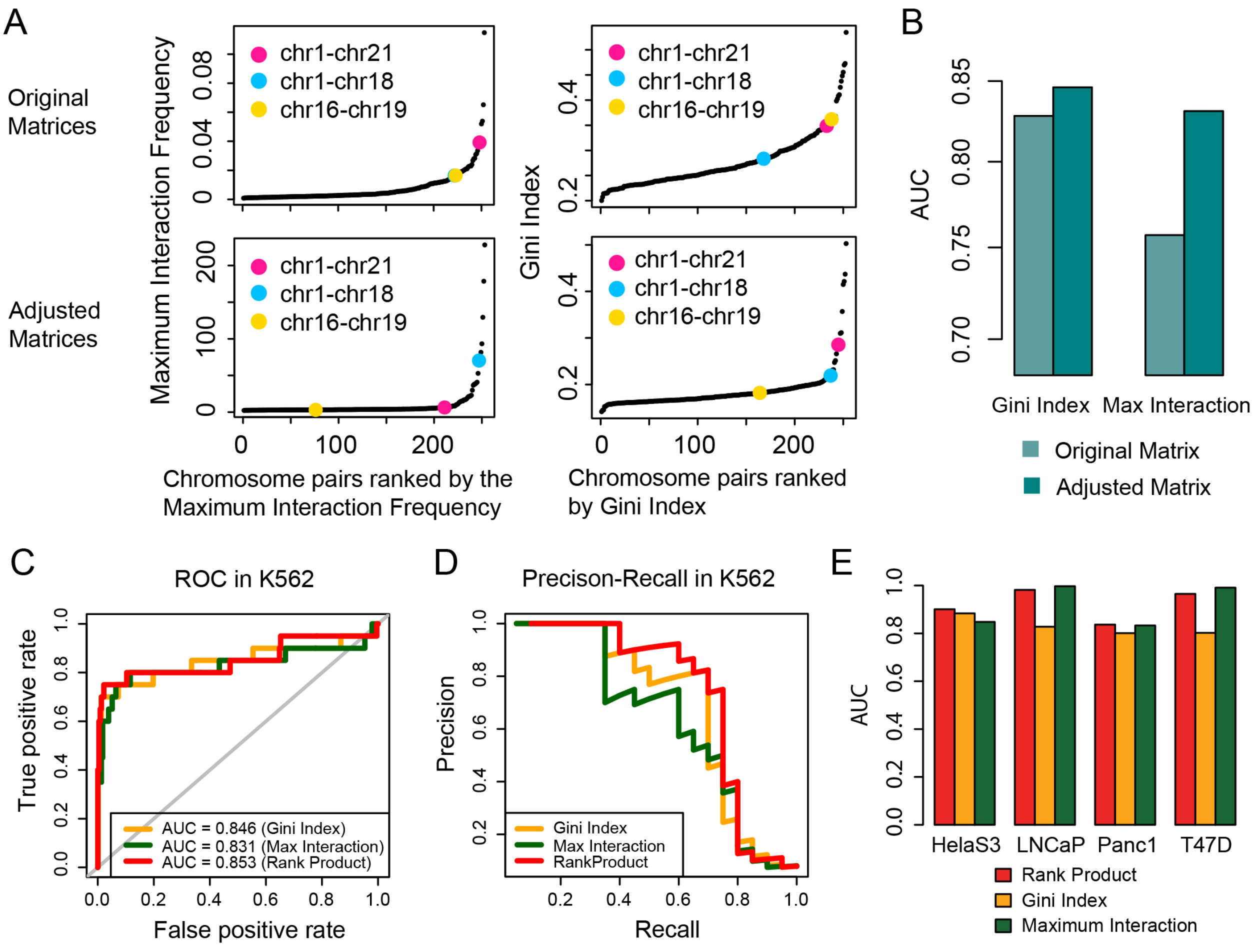

Fig. S12. Rank Product approach accurately identifies translocated chromosome pairs. A, The distribution of the maximum interaction frequency (MIF, left), the Gini Index (right), and the rank product of these two (figure 4A) in inter-chromosome contact matrices before (upper) and after (lower) adjustment in K652 cells. Chromosomal pairs in pink and blue correspond to two FISH-validated translocation pairs (chr1, chr21) and (chr1, chr18); the one in yellow corresponds to a chromosome pair (chr16, chr19) without translocation. **B,** AUROC values show either Gini Index or MIF perform better after the background subtraction in K562 cells. **C-D,** ROC curves (C) and precision-recall curves (D) of translocated chromosomal pairs predicted by using Gini Index only (orange), the maximum interaction only (dark green), and the rank product of these two (red) in K562 cells. E, Performance of rank product, Gini index, and the maximum interactions in HelaS3, LNCaP, Panc1, and T47D cells.

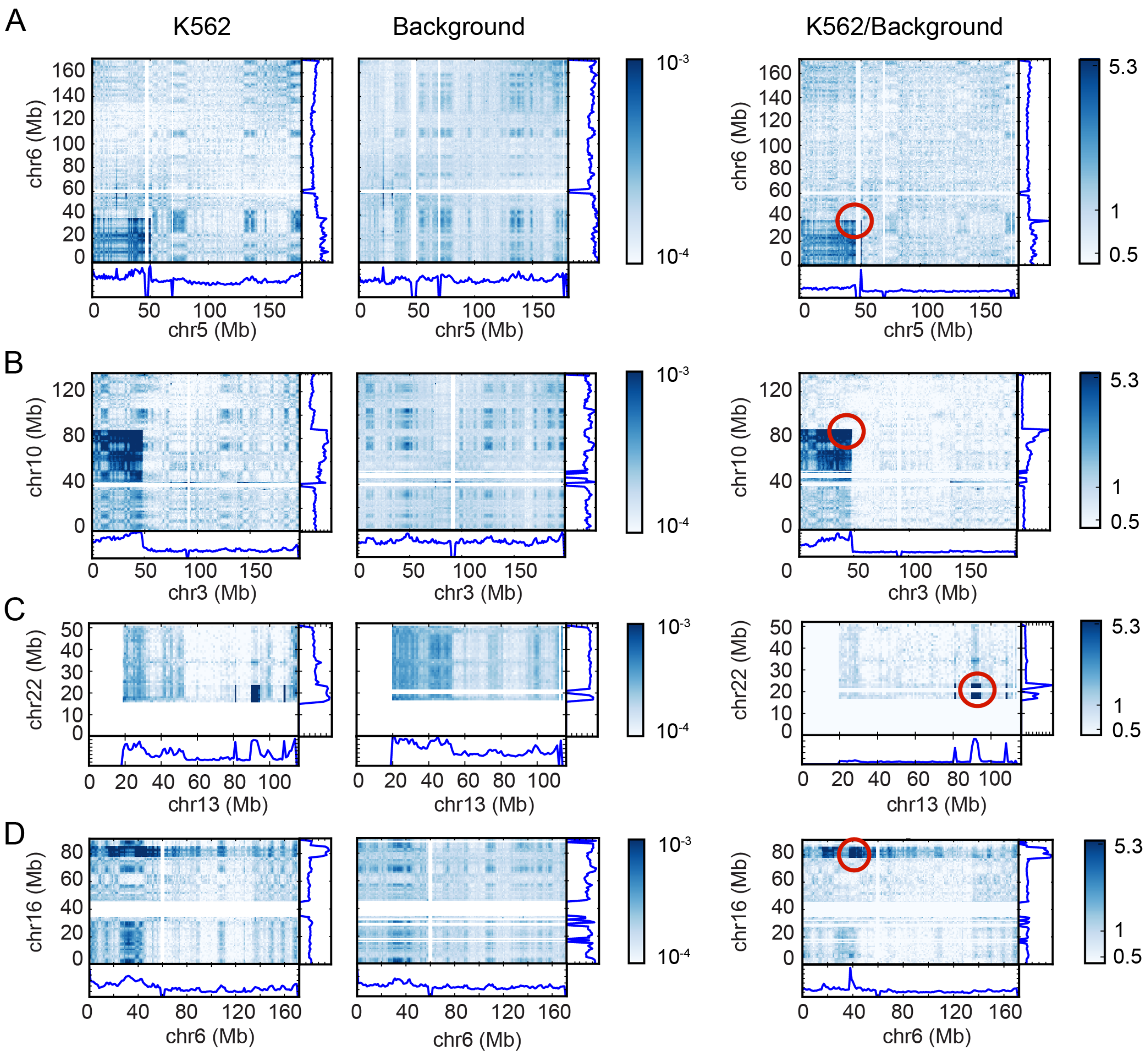

Fig. S13. **Examples of chromosomal pairs with most significant rank product. A-D**, Hi-C inter-chromosomal heatmaps and 1D coverage in original K562(left), background (middle), and adjusted K562 (K562/Background, right) data. Hi-C 1-D profiles (the sum of rows and columns of each inter-chromosomal interaction matrix) are shown along with the interaction maps. Translocation breakpoints are marked by red circles.

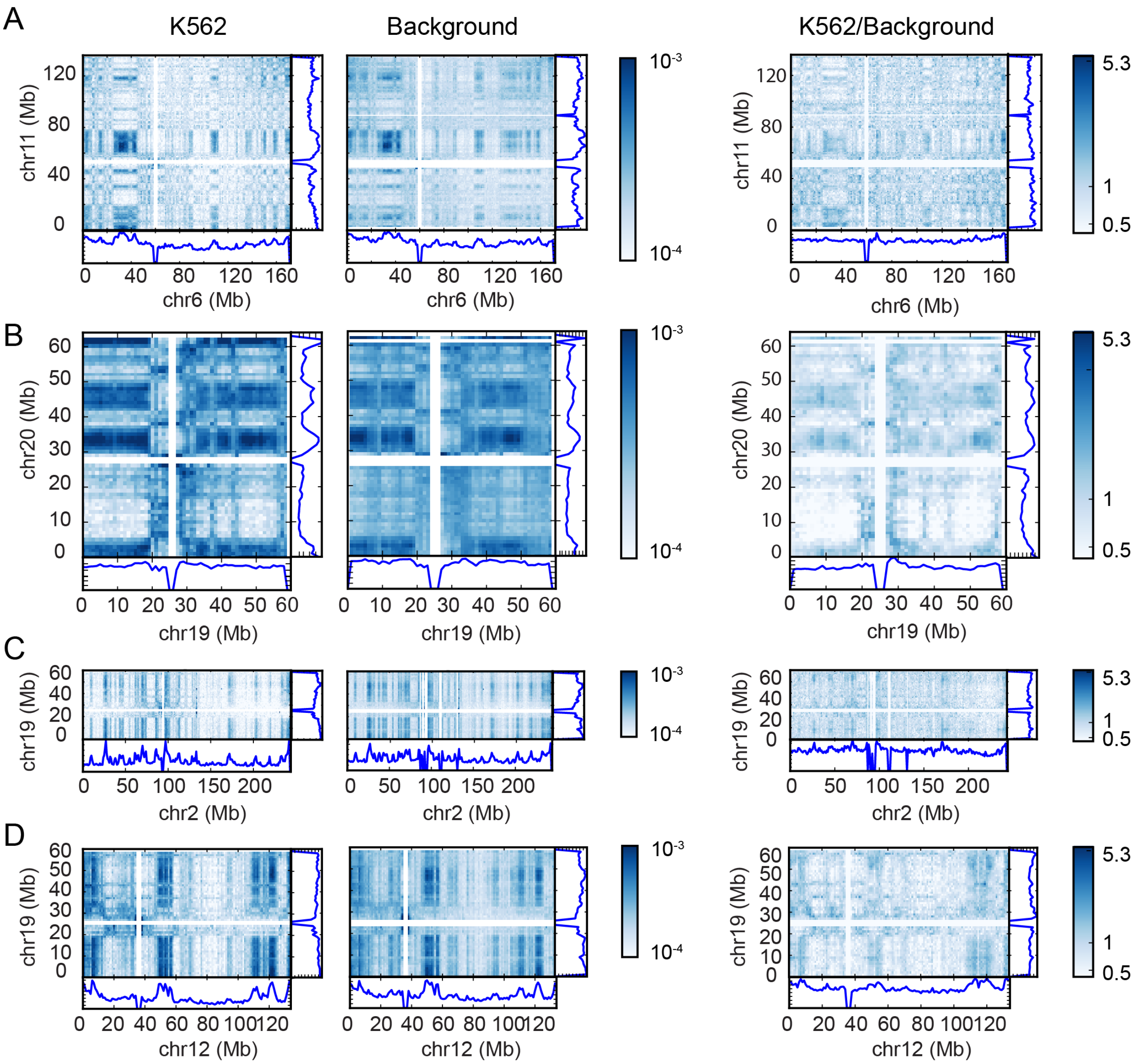

Fig. S14. **Examples of missed translocated chromosomal pairs by HiNT. A-D**, Hi-C inter-chromosomal heatmaps and 1D coverage in original K562(left), background (middle), and adjusted K562 (K562/Background, right) data. Hi-C 1-D profiles (the sum of rows and columns of each inter-chromosomal interaction matrix) are shown along with the interaction maps.

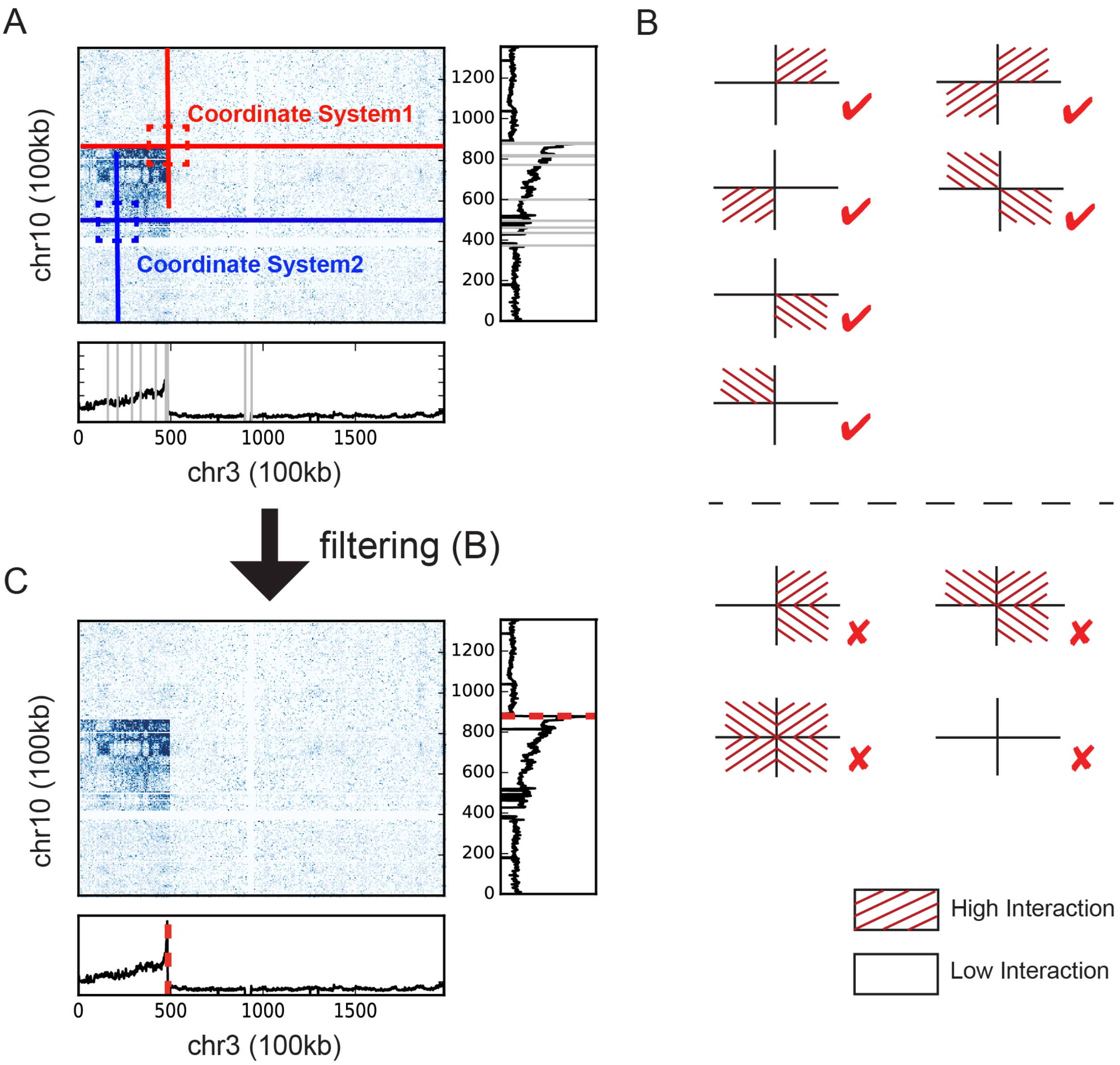

Fig. S15. **Breakpoint detection and filtering. A,** Candidate breakpoints (grey lines) detected by *strucchange* based on the 1D coverage profile (sum of rows and columns). Two-dimensional Cartesian coordinate systems originating from the intersection of each pair of candidate breakpoints are constructed; two examples are shown in the figure. **B,** Patterns of Hi-C interaction frequencies in four 5-bin-by-5-bin quadrants, that generated by the pair of breakpoints from both chromosomes. Valid translocation breakpoints are shown above the dash line, and invalid breakpoints are shown below. **C,** Translocation breakpoints (red dotted lines) after the filtering step.

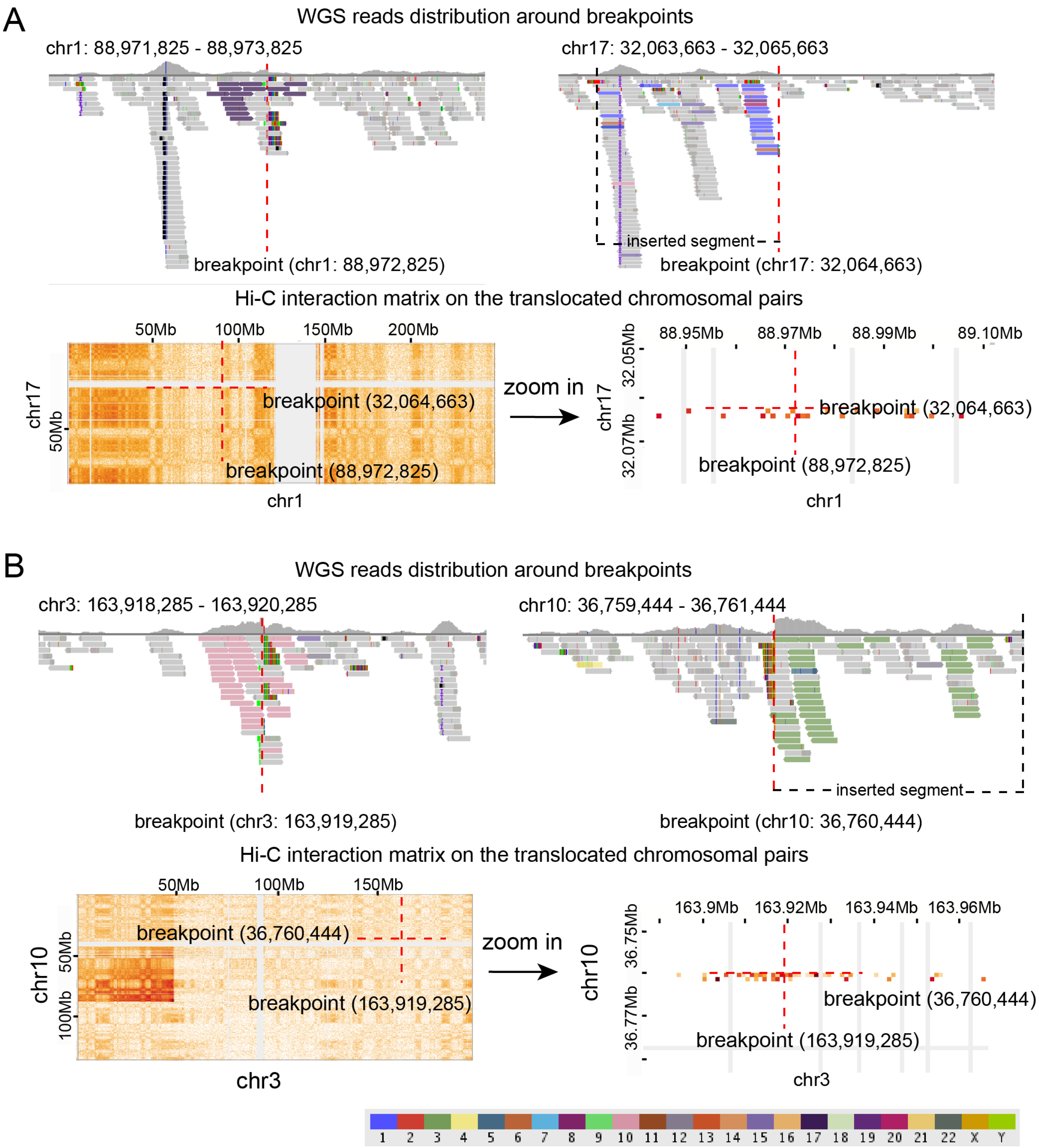

Fig. S16. Examples of the small segment inter-chromosomal insertional translocation that is detected from only WGS. A, The distribution of discordant reads and clipped reads around the translocation breakpoints detected from WGS on chr1 and chr17 (upper); Hi-C interaction heatmap across the whole chromosomes (bottom left) and regions around breakpoints (bottom right). **B,** Similar to A, but the translocation between chr3 and chr10. In the IGV screenshot (WGS reads distribution), each color bar represents a SNV (single nucleotide variant), and the colored reads are paired end reads coded by the chromosome on which their mates can be found. The color code for discordant reads is shown at the bottom.

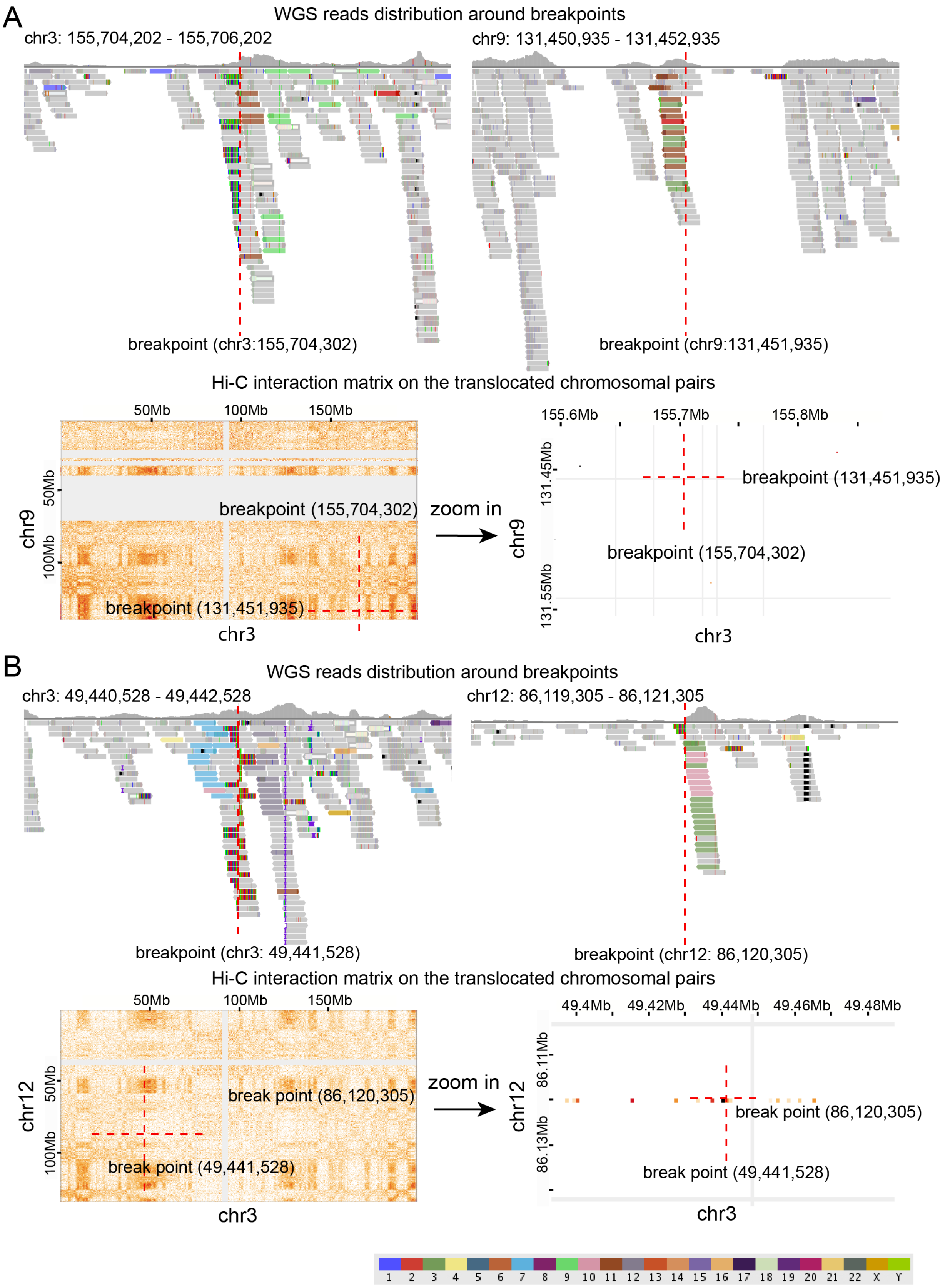

A
WGS reads distribution around breakpoints
chr3: 155,704,202 - 155,706,202
chr9: 131,450,935 - 131,452,935
breakpoint (chr3:155,704,302)
breakpoint (chr9:131,451,935)
Hi-C interaction matrix on the translocated chromosomal pairs
50Mb
100Mb
150Mb
chr9
50Mb
100Mb
breakpoint (155,704,302)
breakpoint (131,451,935)
chr3
zoom in
155.6Mb
155.7Mb
155.8Mb
131.45Mb
131.55Mb
breakpoint (131,451,935)
breakpoint (155,704,302)
chr3
B
WGS reads distribution around breakpoints
chr3: 49,440,528 - 49,442,528
chr12: 86,119,305 - 86,121,305
breakpoint (chr3: 49,441,528)
breakpoint (chr12: 86,120,305)
Hi-C interaction matrix on the translocated chromosomal pairs
50Mb
100Mb
150Mb
chr12
50Mb
100Mb
break point (86,120,305)
break point (49,441,528)
chr3
zoom in
49.4Mb
49.42Mb
49.44Mb
49.46Mb
49.48Mb
86.11Mb
86.13Mb
break point (86,120,305)
break point (49,441,528)
chr3
1 2 3 4 5 6 7 8 9 10 11 12 13 14 15 16 17 18 19 20 21 22 X Y

Fig. S17. Examples of the complex SVs. **A,** The distribution of discordant reads and clipped reads around the translocation breakpoints detected from WGS on chr3 and chr9 (upper); Hi-C interaction heatmap across the whole chromosomes (bottom left) and regions around breakpoints (bottom right). **B,** Similar to A, but the translocation between chr3 and chr12. In the IGV screenshot (WGS reads distribution), each color bar represents a SNV (single nucleotide variant), and the colored reads are paired end reads coded by the chromosome on which their mates can be found. The color code for discordant reads is shown at the bottom.

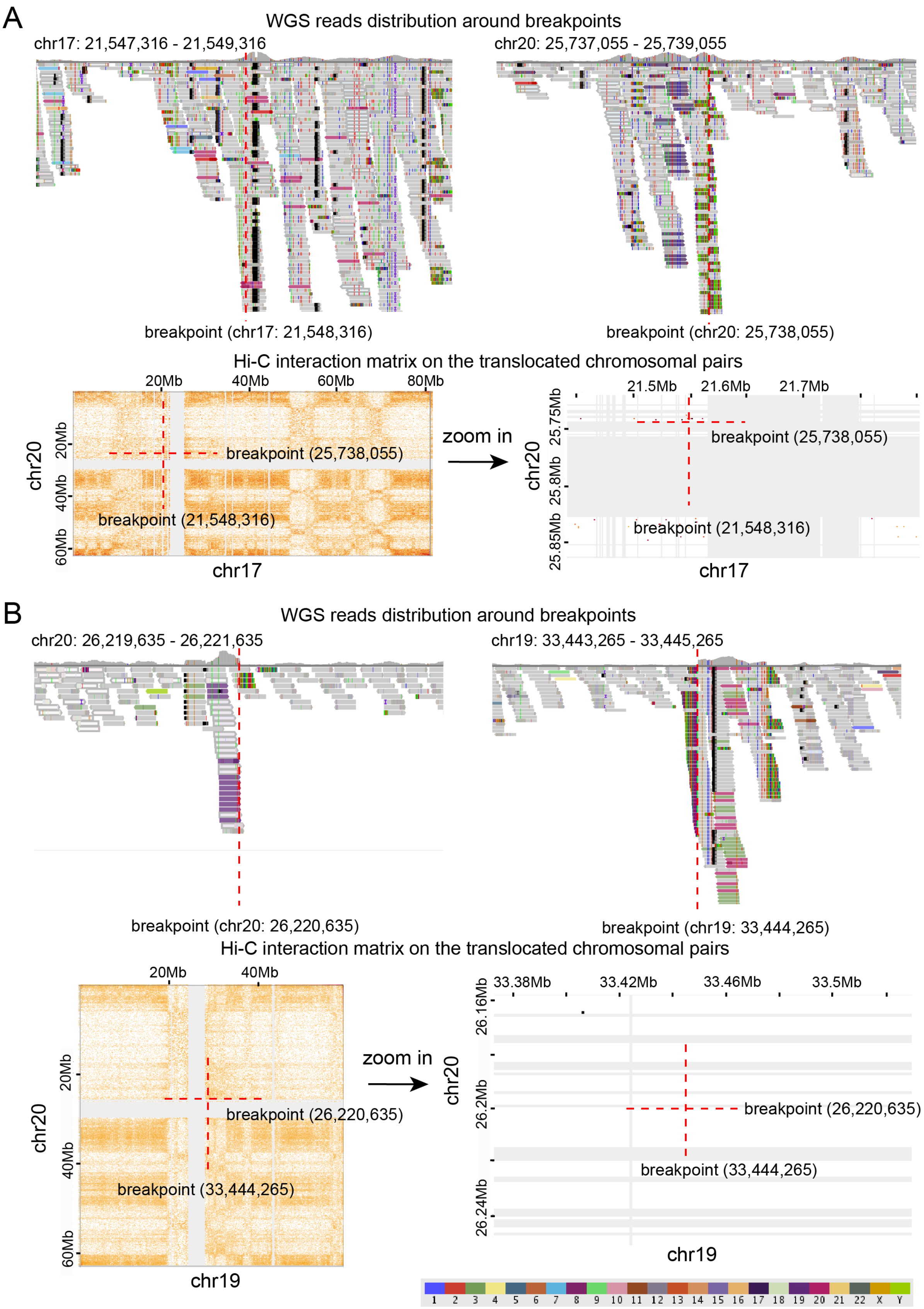
A
WGS reads distribution around breakpoints
chr17: 21,547,316 - 21,549,316
chr20: 25,737,055 - 25,739,055
breakpoint (chr17: 21,548,316)
breakpoint (chr20: 25,738,055)
Hi-C interaction matrix on the translocated chromosomal pairs
20Mb
40Mb
60Mb
80Mb
chr20
20Mb
40Mb
60Mb
breakpoint (25,738,055)
breakpoint (21,548,316)
chr17
zoom in
21.5Mb
21.6Mb
21.7Mb
25.75Mb
25.8Mb
25.85Mb
breakpoint (25,738,055)
breakpoint (21,548,316)
chr17
B
WGS reads distribution around breakpoints
chr20: 26,219,635 - 26,221,635
chr19: 33,443,265 - 33,445,265
breakpoint (chr20: 26,220,635)
breakpoint (chr19: 33,444,265)
Hi-C interaction matrix on the translocated chromosomal pairs
20Mb
40Mb
chr20
20Mb
40Mb
60Mb
breakpoint (26,220,635)
breakpoint (33,444,265)
chr19
zoom in
33.38Mb
33.42Mb
33.46Mb
33.5Mb
26.16Mb
26.2Mb
26.24Mb
breakpoint (26,220,635)
breakpoint (33,444,265)
chr19
1 2 3 4 5 6 7 8 9 10 11 12 13 14 15 16 17 18 19 20 21 22 X Y

Fig. S18. Examples of the false positives that identified from WGS data. A, The distribution of discordant reads and clipped reads around the translocation breakpoints detected from WGS on chr17 and chr20 (upper); Hi-C interaction heatmap across the whole chromosomes (bottom left) and regions around breakpoints (bottom right). **B,** Similar to A, but the translocation between chr19 and chr20. In the IGV screenshot (WGS reads distribution), each color bar represents a SNV (single nucleotide variant), and the colored reads are paired end reads coded by the chromosome on which their mates can be found. The color code for discordant reads is shown at the bottom.

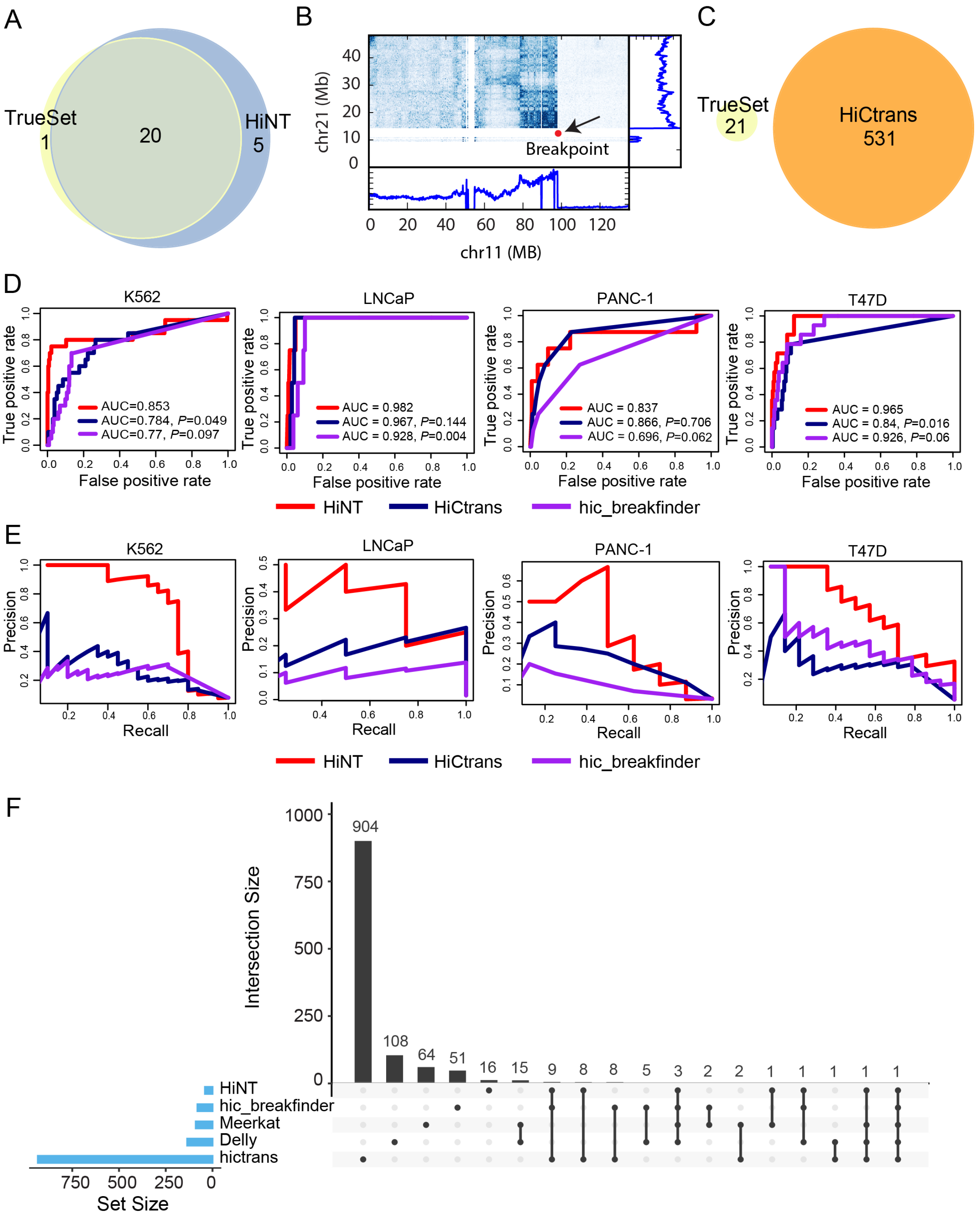
A
TrueSet
1
20
HiNT
5
B
chr21 (Mb)
40
30
20
10
0
Breakpoint
0
20
40
60
80
100
120
chr11 (MB)
C
TrueSet
21
HiCtrans
531
D
K562
True positive rate
False positive rate
AUC=0.853
AUC=0.784, P=0.049
AUC=0.77, P=0.097
LNCaP
AUC = 0.982
AUC = 0.967, P=0.144
AUC = 0.928, P=0.004
PANC-1
AUC = 0.837
AUC = 0.866, P=0.706
AUC = 0.696, P=0.062
T47D
AUC = 0.965
AUC = 0.84, P=0.016
AUC = 0.926, P=0.06
HiNT
HiCtrans
hic_breakfinder
E
K562
Precision
Recall
LNCaP
PANC-1
T47D
HiNT
HiCtrans
hic_breakfinder
F
Intersection Size
1000
750
500
250
0
904
108
64
51
16
15
9
8
8
5
3
2
2
1
1
1
1
1
HiNT
hic_breakfinder
Meerkat
Delly
hictrans
750
500
250
0
Set Size

Fig. S19. **HiNT outperforms existing methods on translocation breakpoints detection in both simulated and real Hi-C data**. **A,** The overlap of translocation breakpoints detected by HiNT and simulated true set. **B,** Hi-C interaction heatmap for the breakpoint that was missed by HiNT, the sum of rows and columns are shown along the matrix. **C,** The overlap of translocation breakpoints detected by HiCtrans and simulated true set. **D-E,** Evaluation of the performance of HiNT (red curve), HiCtrans (navy curve), and hic_breakfinder (purple curve) on translocated chromosome pairs prediction in K562, LNCaP, PANC-1, and T47D cells by ROC curves (D) and precision-recall curves (E). *P-values* (see Methods) for the AUC comparison between HiNT and HiCtrans/OneD are labeled in the figures. **F,** Intersections of the translocation breakpoints detected by Meerkat and Delly from WGS, and HiNT, HiCtrans and hic_breakfinder from Hi-C.
