# Supplementary material for: HiNT: a computational method for detecting copy number variations and translocations from Hi-C data
Source: Genome Biol. 2020 Mar 23;21:73. doi: 10.1186/s13059-020-01986-5 (PMC7087379; doi:10.1186/s13059-020-01986-5)
Supplement: Supplementary file 7 — Review history. [file 13059_2020_1986_MOESM7_ESM.docx]

**First round**

**Reviewer 1**

Authors describe HINT pipeline, which allows for detection of copy number variations and translocations from Hi-C data. The paper is overall well written, though lacking in detail in some sections. Authors validate method performance by using real and simulated data and convincingly show that they have a useful method. Necessary comparisons to most existing methods are performed, showing that HINT largely outperforms other methods at CNV and translocation detection. This is a useful manuscript that can adopted by researchers in the field. I think the paper can benefit from extending some of the analysis to a couple other cell types and better explaining the method.
Major points:
Can the GAM model be used to model biases in an iterative manner, by removing regions with CNVs, and refitting the GAM model to remaining bins? A similar strategy is adopted by Fit-Hi-C in learning the background model where outliers are removed in an iterative process where background model is refined at each iteration.
How is the mappability estimated? This detail is missing in the method section. Since BWA-MEM is used, measuring mappability of a genome segment does not seem trivial.
The GAM based normalization approach is quite similar to HiCNorm (Hu et al.). A cite seems appropriate.
CNV detection performance of HINT in impressive but using only two cell types seems limited. Consider extending the analysis to more cell lines, and maybe include HAP1 (a largely haploid cell type) as a negative control.
I recommend tha the authors compare their method against OneD (Vidal et al. 2018 NAR) as this recent method also measures CNVs.
In detecting inter-chromosomal translocation, authors rightfully point out that interchromosomal interactions are confounded by compartmentalization ( and sub-compartmentalization) effects which may confound the translocation detection method. Authors try to normalize such effects away by using an average matrix from different cell types, but this approach is flawed since compartments can be dynamic across cell types. Can an alternative normalization strategy be used where the effects of compartmentalization be normalized away in a cell type specific manner?
What are the characteristics of the translocations detected by WGS (Meekkat and Delly) but not by HINT, if any? Is there any evidence suggesting these are regions are enriched in certain regions with a particular feature of chromatin organization confound the Hi-C signal? Are they enriched in a particular compartment or a region? I realize the authors already attempted to characterize such discrepancies in pages 9-10 but additional findings can make it more compelling.
Hi-glass URL (http://18.215.251.253) did not work.
Please refer to methods section in the main text when glossing over technical details of the method.
Conclusion can be longer, elaborating on the differences between methods and shortcomings of each method. I think the paper does a thorough evaluation of various methods and approaches and those conclusion can be stressed here in more detail.
Please add more detail to the methods section, e.g. how is the Gini Index calculated exactly? At what resolution? How is the mappability estimated for the GAM? Since a local alignment strategy is used, it is not clear to me how the mappability of a region is estimated. Some important details of the method is not clear to the reader.
Minor
Fig2B has color but it does not code for a variable, just positivity/negativity. Slightly confusing.
Please better annotate the curves on the sides of hi-c matrices in the figures, it is not entirely clear that these curves correspond to the row sums and column sums and the figure legend do not always explain this.
What are the colors in Fig 5C?
Please place the detected and undetected translocation in a box in SuppFig 8 and SuppFig 9

**Reviewer 2**

The authors describe a new method for analyzing Hi-C data to characterize CNVs and SVs in human genomes, particularly in cancer.  Their method appears to be substantially better than WGS and other Hi-C-based approaches, particularly in repetitive regions of the genome.  They also make this tool publicly available with installation instructions. Therefore, I expect this tool to be useful to the community.  My main suggestion is to compare their results to a recent publication in my point 1 below, since I expect this will give much greater insight into the strengths and weaknesses of their methods.
1. Although this publication may not have been available when the authors were performing this analysis, I expect it would be very useful if they would compare their CNV and SV predictions to those detected in K562 recently in this paper https://genome.cshlp.org/content/29/3/472.long, since they used WGS, 10x, mate-pair, karyotyping, and aCGH.
2. The authors state: "For the translocations detected only in WGS, some are missed in Hi-C due to its more uneven genome coverage." What fraction are missed due to its uneven coverage?
3. Could the authors compare the performance they see for other methods like HiCtrans and HiCnv relative to the performance reported in the publications about these tools, and ideally explain any discrepancies?
4. It would be good to perform statistical tests when claiming HINT outperforms other methods, e.g., for the AUC comparisons in line 304.

**Response to reviewers**

**Reviewer 1**

Authors describe HINT pipeline, which allows for detection of copy number variations and translocations from Hi-C data. The paper is overall well written, though lacking in detail in some sections. Authors validate method performance by using real and simulated data and convincingly show that they have a useful method. Necessary comparisons to most existing methods are performed, showing that HINT largely outperforms other methods at CNV and translocation detection. This is a useful manuscript that can adopted by researchers in the field. I think the paper can benefit from extending some of the analysis to a couple other cell types and better explaining the method.

Major points:

Can the GAM model be used to model biases in an iterative manner, by removing regions with CNVs, and refitting the GAM model to remaining bins? A similar strategy is adopted by Fit-Hi-C in learning the background model where outliers are removed in an iterative process where background model is refined at each iteration.

We thank the reviewer for this interesting suggestion. We tested the idea in both K562 and GM12878 cells. The results showed that the GAM model fit much better when we removed the bins with CNVs, as suggested (R^2 improved to 0.916 and 0.901).

|  | # of original bins | # of bins after |  | R-square after |
| --- | --- | --- | --- | --- |
|  | bin size (50 kb) | removing CNVs | Original R-square | removing CNVs |
| K562 | 56955 | 26254 | 0.631 | 0.916 |
| GM12878 | 56955 | 41115 | 0.798 | 0.901 |

However, when we compare the copy ratio calculated from the iterative model, we found that the prediction of CNVs estimated by using the re-fitted model does not improve noticeably compared to the original model. The main reason for this is that in the cell lines we examined, there were either too many or too few CNVs. When there are too many CNVs (e.g., K562), excluding CNV regions removes too much information; when there are too few, it does not change the model substantively. The iterative model should work best in the genomes with the number of copy number variants in some intermediate range, but it was not easy to define exactly what it should be.

K562

GM12878

Consistent with the figure above, the comparison of the CNV segmentation between the original HiNT and the iterative model shows that the iterative model does not improve the prediction in the two cell lines (see figure below).

Therefore, we have now added the option to the code on GitHub, but the analysis we discussed in the paper does not use the iterative model.

How is the mappability estimated? This detail is missing in the method section. Since BWAMEM

is used, measuring mappability of a genome segment does not seem trivial.

The mappability for a bin is simply the average mappability across the positions in a bin (unique reads only). The mappability track was downloaded from the UCSC table browser (https://hgdownload.soe.ucsc.edu/goldenPath/hg19/encodeDCC/wgEncodeMapability/wgEncod

eCrgMapabilityAlign50mer.bigWig). We have now clarified this in the Method section. It was important to have this mappability term in the model for prediction of CNVs from Hi-C data, as (Supp. Fig. 3) and previous studies [1-3] have found that the likelihood of observing Hi-C interactions is strongly correlated with mappability.

The GAM based normalization approach is quite similar to HiCNorm (Hu et al.). A cite seems appropriate.

In HiCNorm, there is a Poisson model for removing bias. We have now cited HiCNorm paper in our revised manuscript.

CNV detection performance of HINT in impressive but using only two cell types seems limited. Consider extending the analysis to more cell lines, and maybe include HAP1 (a largely haploid cell type) as a negative control.

In the revised manuscript, we added four more cancer cells: Caki2 (kidney), LNCaP (prostate), MCF7 (breast), and PANC1 (pancreatic), These cell lines have both WGS and Hi-C data available; two were part of a recent paper and the other two were from the CCLE (Cancer Cell Line Encyclopedia) and ENCODE projects. Instead of HAP1, we used another haploid cell CHM13 for comparison since no WGS data were available for HAP1 (note that read depth alone cannot give information on the absolute copy number). Our analysis using these additional data confirms that HiNT outperforms HiCnv and OneD, especially on identification of large CNV segments. In particular, HiNT makes accurate predictions in cells with a normal karyotype (GM12878, CHM13), in which HiCnv and OneD perform poorly. We show the comparison result in Supp. Fig 6-10, which are also copied below.

Caki2

Supplementary figure 6. Evaluation of copy number inference from Hi-C data in Caki2 cells. A, Correlation of log2 copy ratios in each bin (50kb) detected from WGS and HiNT, HiCnv, and OneD in Caki2. B, Comparison of CNV profiles from Hi-C and WGS after segmentation. Red, green and grey bars represent copy gain(log2 copy ratio > 0.3), copy loss(log2 copy ratio < -0.3), and copy neutral regions (log2 copy ratio between -0.3 and 0.3), respectively. C, The number of CNV segments (categorized by size) detected from Hi-C that are also supported by WGS. Specifically, 84%, 76%, and 49% of the large CNV segments identified by HiNT, HiCnv, and OneD are supported by those from WGS, respectively. The overlap criteria for consistency are shown in Supp. Fig. 5C.

LNCaP

Supplementary figure 7. Evaluation of copy number inference from Hi-C data in LNCaP cells. A, Correlation of log2 copy ratios in each bin (50kb) detected from WGS and HiNT, HiCnv, and OneD in LNCaP. B, Comparison of CNV profiles from Hi-C and WGS after segmentation. Red, green and grey bars represent copy gain(log2 copy ratio > 0.3), copy loss(log2 copy ratio < -0.3), and copy neutral regions (log2 copy ratio between -0.3 and 0.3), respectively. C, The number of CNV segments (categorized by size) detected from Hi-C that are also supported by WGS. Specifically, 79%, 84%, and 54% of the large CNV segments identified by HiNT, HiCnv, and OneD are supported by those from WGS, respectively. The overlap criteria for consistency are shown in Supp. Fig. 5C.

MCF7

Supplementary figure 8. Evaluation of copy number inference from Hi-C data in MCF7 cells. A, Correlation of log2 copy ratios in each bin (50kb) detected from WGS and HiNT, HiCnv, and OneD in MCF7. B, Comparison of CNV profiles from Hi-C and WGS after segmentation. Red, green and grey bars represent copy gain(log2 copy ratio > 0.3), copy loss(log2 copy ratio < -0.3), and copy neutral regions (log2 copy ratio between -0.3 and 0.3), respectively. C, The number of CNV segments (categorized by size) detected from Hi-C that are also supported by WGS. Specifically, 76%, 71%, and 49% of the large CNV segments identified by HiNT, HiCnv, and OneD are supported by those from WGS, respectively. The overlap criteria for consistency are shown in Supp. Fig. 5C.

PANC1

Supplementary figure 9. Evaluation of copy number inference from Hi-C data in PANC-1 cells. A, Correlation of log2 copy ratios in each bin (50kb) detected from WGS and HiNT, HiCnv, and OneD in PANC-1. B, Comparison of CNV profiles from Hi-C and WGS after segmentation. Red, green and grey bars represent copy gain(log2 copy ratio > 0.3), copy loss(log2 copy ratio < -0.3), and copy neutral regions (log2 copy ratio between -0.3 and 0.3), respectively. C, The number of CNV segments (categorized by size) detected from Hi-C that are also supported by WGS. Specifically, 84%, 81%, and 58% of the large CNV segments identified by HiNT, HiCnv, and OneD are supported by those from WGS, respectively. The overlap criteria for consistency are shown in Supp. Fig. 5C.

CHM13

Supplementary figure 10. Evaluation of copy number inference from Hi-C data in CHM13 cells. A, Correlation of log2 copy ratios in each bin (50kb) detected from WGS and HiNT, HiCnv, and OneD in CHM13. B, Comparison of CNV profiles from Hi-C and WGS after segmentation. Red, green and grey bars represent copy gain(log2 copy ratio > 0.3), copy loss(log2 copy ratio < -0.3), and copy neutral regions (log2 copy ratio between -0.3 and 0.3), respectively. C, The number of CNV segments (categorized by size) detected from Hi-C that are also supported by WGS. Specifically, 92%, 71%, and 95% of the large CNV segments identified by HiNT, HiCnv, and OneD are supported by those from WGS, respectively. The overlap criteria for consistency are shown in Supp. Fig. 5C.

I recommend that the authors compare their method against OneD (Vidal et al. 2018 NAR) as

this recent method also measures CNVs.

We thank the referee for the suggestion. We have now included OneD [4] in all our comparisons

(Fig2 and Supp. Fig5-10). See figures we copied above.

In detecting inter-chromosomal translocation, authors rightfully point out that interchromosomal interactions are confounded by compartmentalization (and sub-compartmentalization) effects which may confound the translocation detection method. Authors try to normalize such effects away by using an average matrix from different cell types, but this approach is flawed since compartments can be dynamic across cell types. Can an alternative normalization strategy be used where the effects of compartmentalization be normalized away in a cell type specific manner?

We thank the reviewer for raising this interesting and important point. We do agree that A/B compartmentalization has cell-type specificity. Below, we describe our effort to perform cell type-specific normalization. Unfortunately, we find that while the procedure could remove the A/B compartments in some cases, it could also introduce additional artifacts.

To normalize for this effect in each cell type, we tried the method by Imakaev et al [5], which applies eigenvector decomposition to the inter-chromosomal contact map and can subsequently remove the lower-eigenvalue components that may correspond to compartments. Specifically, the contact 𝑇 between bin 𝑖 and 𝑗 can be generated by the following equation:

*T_ij_ =* Σ_k_λ_k_*E_i_^k^E_j_^k^* + <*T*>

< 𝑇 > is the mean value of the matrix, 𝜆) is the kth eigenvalue, and 𝐸) is the corresponding eigenvector, where 𝑘 runs from 1 to 𝑁.

As suggested in Imakaev’s paper, we used the top three eigenvectors, which are robust enough for the reconstruction of the inter-chromosomal matrices, to reconstruct the inter-chromosomal interactions using the above equation. We show two examples of the reconstructed interchromosomal interaction maps below:

Our data indicate that the re-constructed inter-chromosomal interaction maps (upper-right corner of each subfigure) reflect the A/B compartment patterns well. We then obtain the enrichment matrix (adjusted matrix) by dividing the observed (original) by the expected (reconstructed) matrix. However, we found that although the compartment patterns may be reduced in some interchromosomal interaction maps by the normalization process, it also introduces some bias. In the figure below, we illustrate two different cases. In the top line (chr1-chr20), the A/B compartment was removed successfully, as the lower-left corner that corresponds to the A/B compartment has disappeared in the adjusted matrix. In the second example, however, reduction of the A/B compartment was hardly noticeable. Instead, a new artifactual interactions seems to have appeared (small strip noted in blue).

Given these results, we have decided that the current method of averaging all the Hi-C maps in normal cells as the background is a reasonable approach for now.

What are the characteristics of the translocations detected by WGS (Meekkat and Delly) but not by HINT, if any? Is there any evidence suggesting these are regions are enriched in certain regions with a particular feature of chromatin organization confound the Hi-C signal? Are they enriched in a particular compartment or a region? I realize the authors already attempted to characterize such discrepancies in pages 9-10 but additional findings can make it more compelling.

This is an excellent question. For the translocations detected only in WGS, 6 out of 15 are missed in Hi-C simply due to its low resolution. Due to the nature of the assay, the coverage particularly in the intergenic regions is sparse regardless of the sequencing depth. As an example (Supp. Fig. 16A-B, copied below), when there is a translocation that turns out to be an insertion of a small segment from another chromosome, the Hi-C map does not show strong evidence (indicated by a red dotted lines in the lower-left panel of each of the two subfigures below). When one zooms into that area, there is some interaction indicative of the translocated boundary; however, the interaction is too weak to be detected (without incurring too many false positives).

Two of the other 9 cases appear to be complex SVs. In the two examples we show in Supp. Fig17 (copied below), discordant reads around the breakpoint are from two different chromosomes (as indicated by the two different colors). Regardless of the exact details of the SV, it is clear that the Hi-C map (lower-left panel) does not capture the interactions and thus HiNT cannot detect them.

For the remaining 7 cases, we think these are false positive calls in WGS, often occurring in repetitive regions. We find that the discordant reads from WGS for these cases contain a large fraction of single nucleotide polymorphisms or have low mapping qualities, indicating issues in read alignment (Supp. Fig. 18, copied below). Consistent with those being false positive WGS calls, no translocation-associated features are found in the Hi-C interaction maps.

Examples of small segment inter-chromosomal insertional translocations

Supplementary Figure 16. Examples of the small segment inter-chromosomal insertional translocation that is detected from only WGS. A, The distribution of discordant reads and clipped reads around the translocation breakpoints detected from WGS on chr1 and chr17 (upper); Hi-C interaction heatmap across the whole chromosomes (bottom left) and regions around breakpoints (bottom right). B, Similar to A, but the translocation between chr3 and chr10. In the IGV screenshot (WGS reads distribution), each color bar represents a SNV (single nucleotide variant), and the colored reads are paired end reads coded by the chromosome on which their mates can be found. The color code for discordant reads is shown at the bottom.

Examples of complex SVs

Supplementary Figure 17. Examples of the complex SVs. A, The distribution of discordant reads and clipped reads around the translocation breakpoints detected from WGS on chr3 and chr9 (upper); Hi-C interaction heatmap across the whole chromosomes (bottom left) and regions around breakpoints (bottom right). B, Similar to A, but the translocation between chr3 and chr12. In the IGV screenshot (WGS reads distribution), each color bar represents a SNV (single nucleotide variant), and the colored reads are paired end reads coded by the chromosome on which their mates can be found. The color code for discordant reads is shown at the bottom.

Examples of false positives

Supplementary Figure 18. Examples of the false positives that identified from WGS data. A, The distribution of discordant reads and clipped reads around the translocation breakpoints detected from WGS on chr17 and chr20 (upper); Hi-C interaction heatmap across the whole chromosomes (bottom left) and regions around breakpoints (bottom right). B, Similar to A, but the translocation between chr19 and chr20. In the IGV screenshot (WGS reads distribution), each color bar represents a SNV (single nucleotide variant), and the colored reads are paired end reads coded by the chromosome on which their mates can be found. The color code for discordant reads is shown at the bottom.

Hi-glass URL (http://18.215.251.253) did not work.

We have removed the hi-glass page since it is redundant with the supplementary table 4.

Please refer to methods section in the main text when glossing over technical details of the method.

Following the reviewer’s advice, we have referred to the Methods section at the appropriate places in the main text.

Conclusion can be longer, elaborating on the differences between methods and shortcomings of each method. I think the paper does a thorough evaluation of various methods and approaches and those conclusion can be stressed here in more detail.

We have lengthened the discussion of differences between methods in each part of the evaluation to emphasize our findings. The conclusion section is also refined to accommodate those changes.

Please add more detail to the methods section, e.g. how is the Gini Index calculated exactly? At what resolution? How is the mappability estimated for the GAM? Since a local alignment strategy is used, it is not clear to me how the mappability of a region is estimated. Some important details of the method is not clear to the reader.

We have added more details to the methods section, including the following:

Gini Index calculation

For each Hi-C inter-chromosomal interaction matrix M (at 1Mb resolution), we first sorted the contact regions from lowest to highest, based on the adjusted contact frequencies between these two regions. Regions that did not form contacts with any other regions were excluded. The cumulative contact frequencies for the sorted regions is the Lorenz curve. The Gini index is a measure of how much this curve deviates from the diagonal, and, in its simplified form, is computed as twice the area between the Lorenz curve and the diagonal.

Mappability used in GAM

We downloaded the mappability track from the UCSC table browser

(https://hgdownload.soe.ucsc.edu/goldenPath/hg19/encodeDCC/wgEncodeMapability/wgEncod

eCrgMapabilityAlign50mer.bigWig). The bigwig format is then converted to the bedgraph format and used in the GAM model.

Minor

Fig2B has color but it does not code for a variable, just positivity/negativity. Slightly confusing.

We think that using different colors for positive/negativity makes it easier to compare between profiles. We now describe the colors in the figure inset as well: segments with log copy ratio larger than 0.3 as in red; less than -0.3 as in dark green; and copy ratios between -0.3 and 0.3 in grey.

Please better annotate the curves on the sides of hi-c matrices in the figures, it is not entirely clear that these curves correspond to the row sums and column sums and the figure legend do not always explain this.

We now describe the curves on the sides on Hi-C matrices in the figure legend.

What are the colors in Fig 5C?

The colored reads are the discordant reads; exactly to which chromosomes they map is not relevant for illustrating the algorithm performance. However, some readers may not be familiar with this, and so we have now included the color code in the figure.

Please place the detected and undetected translocation in a box in SuppFig 8 and SuppFig 9

We have placed the detected translocations in circles (SuppFig 8 is now SuppFig 13 in the revised manuscript). For SuppFig 9, now SuppFig 14, we obtained the translocation information from a previously published K562 karyotype [7, 8] and do not have the exact coordinates.

**Reviewer 2**

The authors describe a new method for analyzing Hi-C data to characterize CNVs and SVs in human genomes, particularly in cancer. Their method appears to be substantially better than WGS and other Hi-C-based approaches, particularly in repetitive regions of the genome. They also make this tool publicly available with installation instructions. Therefore, I expect this tool to be useful to the community. My main suggestion is to compare their results to a recent publication in my point 1 below, since I expect this will give much greater insight into the strengths and weaknesses of their methods.

1. Although this publication may not have been available when the authors were performing this analysis, I expect it would be very useful if they would compare their CNV and SV predictions to those detected in K562 recently in this paper https://genome.cshlp.org/content/29/3/472.long, since they used WGS, 10x, mate-pair, karyotyping, and aCGH.

We thank the reviewer for the suggestion. We have downloaded the log2 copy ratios for K562 provided in the GR paper [9]. The paper indicated that the array CGH has consistent results with WGS, and provided the log2 copy ratios only for the array CGH data. We have now added the array CGH-based CNV profile to the list of profiles being compared in Figure 2 (WGS data; HiNT-CNV, HiCnv, and OneD for Hi-C data; OneD is another computational tool suggested by Reviewer 1). We typically see more smooth CNV profiles at lower resolution for array CGH data, but that was not the case for the log-ratios computed in that paper. The new Figure 2 is copied below. Since SVs provided in the GR paper include only deletions (DEL), inversions (INV), duplications (DUL), and unknown types (UNK), and HiNT-TL can only predict translocations, we did not perform additional analysis for SVs.

2. The authors state: "For the translocations detected only in WGS, some are missed in Hi-C due to its more uneven genome coverage." What fraction are missed due to its uneven coverage?

Please see our response to reviewer1 on pages 10-13.

3. Could the authors compare the performance they see for other methods like HiCtrans and HiCnv relative to the performance reported in the publications about these tools, and ideally explain any discrepancies?

This is an excellent question. For CNVs, what we have found in our evaluations is that the performance of HiCnv is uneven across datasets. For some cell lines (e.g., LNCaP), it performed reasonably well; for others (e.g., K562, GM12878), it did not perform well. One difference for the good performance group is that they have low sequencing coverage, but it is not clear why the coverage should make a substantial difference.

For translocations, we could not reproduce the results of HiCtrans when we applied their algorithm to some of the datasets used in that paper. Our guess is that they used additional filtering to get the final results, but they neglected to describe the details in their paper. In our manuscript, we used the previously reported FISH-validated translocations as the gold standard to evaluate algorithms, as well as WGS-identified breakpoints.

4. It would be good to perform statistical tests when claiming HINT outperforms other methods, e.g., for the AUC comparisons in line 304.

We have added p-values for the AUC comparisons between HiNT and other methods. The pvalues are calculated with the R package pROC by adopting the bootstrap method [11].

**REFERENCE**

1. Wu H-J, Michor F: **A computational strategy to adjust for copy number in tumor Hi-C**

**data**. *Bioinformatics* 2016:1–7.

2. Yaffe E, Tanay A: **Probabilistic modeling of Hi-C contact maps eliminates systematic**

**biases to characterize global chromosomal architecture.** *Nat Genet* 2011, **43**:1059–1065.

3. Chakraborty A, Ay F: **Identification of copy number variations and translocations in**

**cancer cells from Hi-C data.** *Bioinformatics* 2017, **34**:338–345.

4. Vidal E, le Dily F, Quilez J, Stadhouders R, Cuartero Y, Graf T, Marti-Renom MA, Beato M,

Filion GJ: **OneD: increasing reproducibility of Hi-C samples with abnormal karyotypes**.

*Nucleic Acids Research* 2018, **46**:e49–e49.

5. Imakaev M, Fudenberg G, McCord RP, Naumova N, Goloborodko A, Lajoie BR, Dekker J,

Mirny LA: **Iterative correction of Hi-C data reveals hallmarks of chromosome organization**.

*Nat Meth* 2012, **9**:999–1003.

19

6. Tubio JMC, Li Y, Ju YS, Martincorena I, Cooke SL, Tojo M, Gundem G, Pipinikas CP,

Zamora J, Raine K, Menzies A, Roman-Garcia P, Fullam A, Gerstung M, Shlien A, Tarpey PS,

Papaemmanuil E, Knappskog S, Van Loo P, Ramakrishna M, Davies HR, Marshall J, Wedge

DC, Teague JW, Butler AP, Nik-Zainal S, Alexandrov L, Behjati S, Yates LR, Bolli N, et al.:

**Extensive transduction of nonrepetitive DNA mediated by L1 retrotransposition in cancer**

**genomes**. *Science* 2014, **345**:1251343–1251343.

7. Taberlay PC, Achinger-Kawecka J, Lun ATL, Buske FA, Sabir K, Gould CM, Zotenko E, Bert

SA, Giles KA, Bauer DC, Smyth GK, Stirzaker C, O'Donoghue SI, Clark SJ: **Three-dimensional**

**disorganization of the cancer genome occurs coincident with long-range genetic and**

**epigenetic alterations.** *Genome Research* 2016, **26**:719–731.

8. Naumann S, Reutzel D, Speicher M, Decker HJ: **Complete karyotype characterization of**

**the K562 cell line by combined application of G-banding, multiplex-fluorescence in situ**

**hybridization, fluorescence in situ hybridization, and comparative genomic hybridization.**

*Leuk Res* 2001, **25**:313–322.

9. Zhou B, Ho SS, Greer SU, Zhu X, Bell JM, Arthur JG, Spies N, Zhang X, Byeon S, Pattni R,

Ben-Efraim N, Haney MS, Haraksingh RR, Song G, Ji HP, Perrin D, Wong WH, Abyzov A,

Urban AE: **Comprehensive, integrated, and phased whole-genome analysis of the primary**

**ENCODE cell line K562**. *Genome Research* 2019, **29**:472–484.

10. Chakraborty A, Ay F: **Identification of copy number variations and translocations in**

**cancer cells from Hi-C data.** *Bioinformatics* 2017.

11. Robin X, Turck N, Hainard A, Tiberti N, Lisacek F, Sanchez J-C, Müller M: **pROC: an opensource**

**package for R and S+ to analyze and compare ROC curves**. 2011:1–8.

**Second round**

**Reviewer 1**

I find the authors' responses to my criticism satisfactory and the revised manuscript very improved. While the authors counterpointers' for retaining certain parts of their manuscript - as opposed to the newer approaches they tried at my request - are valid and reasonable, I have a few minor suggestions to make to the text.

I am afraid the mappability estimation used in the manuscript is not entirely valid. Unless I am msising a detail here, authors use BWA-MEM local alignment strategy, which will result in alignments of different length. However, ENCODE50mer track measures the mappability/uniqueness given a fixed alignemnt length of 50mer. Unfortunately, to the best of my knowledge, measuring the mappability for a local alignment of non-fixed length and using that as a parameter is a challenging and open problem. I have two recommendations to the authors, use a shorter mappability track as opposed to 50bp as a more conservative measure, assuming a large majority of their bwa-mem alignments are, say, ~30bp long, use 36mer track from ENCODE. Otherwise, clarify this the text in methods and recommend the users the method to take care in choosing which track to use. My guess is that using 30 or 50bp mappability tracks will not change the results significantly. Clarification of this in the text would still be helpful.

I thank the authors for analyzing more cell types. I believe the poor quality of LnCAP may be due to the low quality of the Hi-C data. Yardimci et al. 2019 points this fact out comprehensively, since they analyzed the same ENCODE LnCAP data. Authors can point out the reason for the poor performance of LnCAP if they wish so.

Again, the authors did a very nice job trying to remove compartment effects using eigendecomposition. Since the results were not as helpful as we hoped for, I recommend pointng out shortcoming of using the original averaging approach in text, or at least point out the possibility of using more cell type specific averages in the fugure (as an example, built from data from N cell types from same tissue).

The analysis of discprepancies of the between WGS vs. HI-C detected CNVs is quite useful. I recommend adding the insights from these analyses in a bit more detail to conclusion, though I leave it at the authors' discretion.

**Reviewer 2**

The authors have done a good job responding to the reviewer's comments, including a number of new comparisons and analyses
